# **Gut microbiota butyrate mediated RUNX3 promotes Nr4a1**^**high**^**ZFP36**^**high**^ **resident macrophages via NR4A1/ERK1/2 MAPK to maintain gut homeostasis**

**DOI:** 10.1080/19490976.2025.2569741

**Published:** 2025-10-17

**Authors:** Yunhuan Gao, Yi Shi, Ningning Zhu, Yang Hao, Juanjuan Wang, Yuan Zhang, Rongcun Yang

**Affiliations:** a Department of Immunology, Nankai University School of Medicine, Nankai University, Tianjin, China; b School of Statistics and Data Science, Shanghai University of International Business and Economics, Shanghai, China; c State Key Laboratory of Medicinal Chemical Biology, Nankai University, Tianjin, China; d Translational Medicine Institute, Tianjin Union Medical Center of Nankai University, Tianjin, China

**Keywords:** Gut microbe, resident macrophage, RUNX3, Nr4a1, ZFP36

## Abstract

Gut homeostasis is critical for systemic health, and its disruption is implicated in various diseases, including metabolic disorders. Gut-resident macrophages play a pivotal role in maintaining intestinal homeostasis, yet the mechanisms underlying their differentiation and function remain incompletely understood. Using single-cell RNA sequencing (scRNA-seq), we found a key regulatory axis in which gut microbiota-derived butyrate induces the differentiation of Nr4a1^high^ZFP36^high^ resident macrophages via RUNX3 to sustain gut homeostasis. Butyrate could upregulate RUNX3 expression in gut-resident macrophages through lncRNA lncLy6c-mediated H3K4me3 modification. Genetic ablation of RUNX3 in myeloid cells of *RUNX3*^*fl/fl-Lyz2-Cre*^ mice led to a marked reduction in resident macrophages and increased susceptibility to DSS-induced colitis. RUNX3-mediated resident macrophages exhibited elevated ZFP36 expression alongside suppressed pro-inflammatory cytokines and chemokines. Further mechanistic studies revealed that RUNX3 drives the differentiation of Nr4a1^high^ZFP36^high^ resident macrophages via the Nr4a1-dependent activation of the ERK1/2 MAPK pathway. Consistently, high expression levels of RUNX3, Nr4a1, and ZFP36 were observed in colon-resident macrophages from healthy human donors. Collectively, our findings demonstrate that butyrate-RUNX3 signaling orchestrates the differentiation of Nr4a1^high^ZFP36^high^ resident macrophages through the Nr4a1/ERK1/2 MAPK pathway, thereby safeguarding gut homeostasis.

## Graphical abstract

**Figure:**
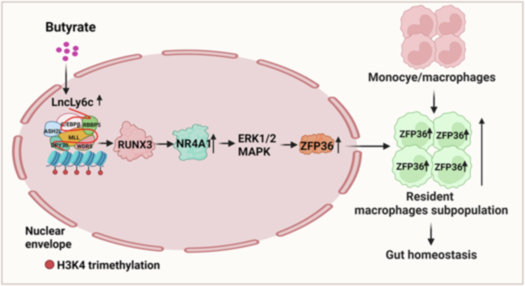

## Introduction

Gut homeostasis is critically important for human health, and its disruption has been associated with numerous diseases including metabolic disorders (obesity, diabetes, and non-alcoholic fatty liver disease), gastrointestinal diseases (inflammatory bowel diseases (IBD) and colorectal cancer (CRC)), as well as neurodegenerative and cardiovascular diseases.^1-3^ The intestinal mucosa harbors the largest population of resident macrophages in both humans^4^ and mice,^5^ which play a pivotal role in maintaining gut homeostasis.^6^^,^^7^ However, the precise mechanisms governing the differentiation and function of these resident macrophages remain incompletely understood.

Gut resident macrophages exhibit a dual origin, arising from both embryonically derived macrophages and peripheral blood inflammatory monocytes, with the latter serving as the primary source of macrophage turnover in adult mice.^8^ Through a process termed the "monocyte waterfall",^8^^,^^9^ circulating inflammatory monocytes migrate to the gut and differentiate into resident macrophages. These monocyte-derived precursors initially express high levels of CCR2, F4/80 (F10), and Ly6C, along with moderate to low levels of cysteine-X3-cysteine chemokine receptor 1 (CX3CR1). Upon tissue integration, they undergo phenotypic maturation, downregulating CCR2, F4/80, and Ly6C while upregulating CX3CR1 and CD206, ultimately adopting the signature profile of CD45^+^CD11b^+^CD64^+^CD103^−^MHCII^+^ gut-resident macrophages.^10-13^ This differentiation process is tightly regulated by the nuclear receptor Nr4a1.^14^^,^^15^ Once established, gut-resident macrophages perform essential homeostatic functions, including bacterial clearance, removal of senescent cells, tissue repair, extracellular matrix remodeling, epithelial renewal, and maintenance of gut motility and immune tolerance.^6^^,^^7^^,^^16^ Their depletion or functional impairment leads to severe pathological consequences, such as submucosal vascular abnormalities, enteric neuron loss, vascular leakage, impaired intestinal motility, and chronic inflammation.^17^^,^^18^ Recent advances in single-cell RNA sequencing (scRNA-seq) have further revealed significant heterogeneity among gut-resident macrophages, identifying multiple distinct subpopulations with unique transcriptional profiles.^19-21^

The gut microbiota plays a pivotal role in the differentiation and functional development of intestinal macrophages.^8^^,^^22^ Studies using germ-free (GF) mice or antibiotic-treated mice with microbiota depletion have demonstrated a significant reduction in monocyte-derived resident macrophages.^8^^,^^22^ Substantial evidence highlights the critical influence of gut microbiota and their metabolic byproducts on macrophage differentiation.^1^^,^^23^ Through single-cell RNA sequencing (scRNA-seq) analysis, we have found that microbiota-derived butyrate promotes the differentiation of Nr4a1^high^ZFP36^high^ resident macrophages via RUNX3, thereby maintaining intestinal homeostasis. Notably, these Nr4a1^high^ZFP36^high^ resident macrophages exhibit elevated expression of the anti-inflammatory factor ZFP36.

## Materials and methods

Reagents and oligoes used in study were listed in on Table S1.

### **Mice**

Four-to six-week-old male or female C57BL/6 mice were obtained from the Nanjing Animal Center (Nanjing, China). RUNX3 macrophage conditional knockout (RUNX3^fl/fl-Lyz2-Cre^) mice and their control littermates (RUNX3^fl/fl^) were generated by the Nanjing Animal Center. LncLy6C-deficient mice^24^ on a C57BL/6J background were generated by the Model Animal Research Center of Nanjing University (Nanjing, Jiangsu, China) using CRISPR-Cas9 system. Cas9 mRNA and sgRNA were co-injected into zygotes. sgRNA direct Cas9 endonuclease cleavage in upstream of exon 1 of *LncLy6C* and downstream of exon 2 of *LncLy6C*, and create a double-strand break. All mice were bred and maintained under specific pathogen-free (SPF) conditions in the Animal Center of Nankai University. All animal procedures were approved by the Institute's Animal Ethics Committee of Nankai University and conducted in accordance with the guidelines of the Institutional Animal Care and Use Committee of the Model Animal Research Center. Experimental variables, including environmental influences, parental genotypes, and husbandry, were strictly controlled.

### **Preparation of macrophages**

For generation of bone marrow-derived macrophages (BMDMs), bone marrow cells (BMCs) were cultured in medium containing macrophage colony-stimulating factor (M-CSF; 30 ng/mL) for 4 d to differentiate into BMDMs.

For analysis of CD117–CD11b + CD115 + Ly6C + cells, the differentiation status of BMCs was assessed by flow cytometry analysis of CD117–CD11b + CD115 + Ly6C + cells.

For generation of human monocyte-derived macrophages (HMDMs), fresh whole blood was collected from healthy volunteers at the Tianjin Blood Center (Tianjin). Peripheral blood mononuclear cells (PBMCs) were isolated from the blood on the day of collection using Ficoll-Paque density gradient centrifugation (Solarbio) according to the manufacturer's instructions. Isolated PBMCs were then cultured in medium supplemented with M-CSF (500 U/mL) for 5 d to generate HMDMs for subsequent ex vivo stimulation.

### **Colon resident macrophage isolation, treatment and flow cytometry**

For the isolation of colon-resident macrophages, a previously established protocol was utilized for both cell isolation and flow cytometry analysis.^25^ Briefly, colons were excised from mice and immediately placed onto PBS (phosphate-buffered saline; without calcium or magnesium; room temperature)-soaked laboratory tissue paper. To isolate lamina propria (LP) lymphocytes, cells were resuspended in 10 mL of the 40% fraction of a Percoll density gradient (40:80) and carefully overlaid onto 5 mL of the 80% fraction in a 15 mL Falcon tube, followed by centrifugation. LP lymphocytes were harvested from the Percoll gradient interphase, stained with antibodies, and sorted via flow cytometry. Colon-resident macrophages (CD45^+^CD11b^+^CD64^+^CD103^–^MHCII^+^Ly6C^–^/low)^10-13^ were identified and collected. Isolated resident macrophages were subsequently treated with siRNA or inhibitors and analyzed.

Following Percoll gradient separation and collection from the interphase, LP lymphocytes were stained for flow cytometric analysis. Dead cells were excluded using 7-AAD staining. For intracellular cytokine staining, cells were stimulated for 6 hours with 50 ng/mL phorbol 12-myristate 13-acetate (PMA) and 1 μg/mL ionomycin in the presence of GolgiStop. Cells were then stained with FITC-, PE-, APC-, or PerCP/Cy5.5-conjugated antibodies. Gating strategy was performed as described in our previously reported method.^26^

### **Mouse models**

For DSS-induced colitis model, DSS-induced colitis was established as previously described.^27^ Briefly, mice received 2.5% DSS (40 kDa; MP Biomedicals) or the indicated concentration in their drinking water *ad libitum* for 7 d, followed by a return to regular drinking water. Body weight was measured every other day. Percent weight change was calculated as: (weight at day X – weight at day 0)/weight at day 0 × 100. Mice were also monitored daily for rectal bleeding, diarrhea, and general signs of morbidity (e.g., hunched posture, failure to groom). Disease activity index (DAI) and histological evaluation were performed according to established methods.^26^^,^^28^ DAI was calculated as the average score of three parameters. Weight loss: 0 = none; 1 = 1%–5%; 2 = 5%–10%; 3 = 10%–15%; 4 = >15%; bleeding: 0 = normal; 2 = slight bleeding; 4 = gross bleeding; diarrhea (stool consistency): 0 = normal; 2 = loose stools; 4 = watery diarrhea. Histology scores were determined as the sum of epithelial (E) and inflammatory infiltrate (I) scores. Inflammatory Infiltrate (I): 0 = no infiltrate; 1 = infiltrate around crypt bases; 2 = infiltrate reaching the lamina muscularis mucosae; 3 = extensive infiltration reaching the lamina muscularis mucosae with mucosal thickening and abundant edema; 4 = infiltration of the lamina submucosa; Epithelium (E): 0 = normal morphology; 1 = goblet cell loss; 2 = goblet cell loss in large areas; 3 = crypt loss; 4 = crypt loss in large areas.

For macrophage transplantation experiments, monocytes/macrophages were isolated from bone marrow cells (BMCs) of the mice. After treatment with shZFP36-, ZFP36-, shNr4a1-, or Nr4a1-expressing lentiviral, these modified monocytes/macrophages were mixed with untreated BMCs and transplanted into lethally irradiated (8Gy single dose; Shepherd Mark I Cesium Irradiator, J.L. Shepherd and Associates) recipient mice. Three weeks post-transplantation, DSS colitis model was induced in the recipient mice.

For ERK1/2 inhibition study, to investigate the potential role of ERK1/2 in resident macrophage differentiation, mice received intraperitoneal injections of an ERK1/2 inhibitor (5 mg/kg; MCE, USA). The inhibitor was administered every other day for a total of five doses. Control mice received an equivalent volume of 0.9% saline.

For butyrate treatment model**,** mice were administered sodium butyrate (NaB; 100 mg/kg) daily via oral gavage for 7 d.

### **Single-cell RNA-Seq processing**

Immune cells isolated from mouse colon tissues were resuspended in 10 mL of a 40% Percoll solution and overlaid onto 5 mL of an 80% Percoll solution in a 15-mL Falcon tube. The gradient was centrifuged at 1,800 rpm (approximately 500 ×g) for 20 min at room temperature. Lymphocytes were collected, stained for sorting, and subjected to single-cell RNA sequencing (scRNA-seq). scRNA-seq processing was performed essentially as described previously.^29^

#### **Single-cell RNA-Seq data analyses**

Single-cell RNA-Seq data were analyzed following a previously described method.^29^ Low-quality cells and doublets were filtered out by removing cells exhibiting >15% mitochondrial gene content or expressing either fewer than 200 genes or more than 6,000 genes. Clustering was performed using the Deep Embedding algorithm for Single-cell Clustering (DESC). Resident macrophages were subsequently subclustered from the initial results. Within each cluster, differentially expressed genes (DEGs) were identified using the Poisson Generalized Linear Model implemented in Seurat. DEGs for each cluster were defined as genes with a log fold-change > 0 and Bonferroni-adjusted *p*-values < 0.05. These DEGs were used for downstream analyses. Enrichment analysis was conducted using Metascape (http://metascape.org/). Pseudotime trajectory analysis of all macrophages was performed using Monocle 2. Violin plots and feature plots were generated using the VlnPlot and FeaturePlot functions, respectively, in Seurat v4. Intercellular communication was inferred from the scRNA-seq data using CellChat.^30^ Volcano plots were used to visualize gene expression differences. Differential gene expression comparisons between macrophage populations were performed using Seurat, applying a *p*-value cutoff of 10e−3 and log2 (fold-change) cutoff between 1 and –1.

### **RNA-seq analysis**

RNA-seq analyses were done according to reported method.^31^ According to the manual of TRIzol®reagent (Invitrogen, Shanghai, China), total RNAs from cells were extracted. Library was prepared and transcriptome was sequenced on an MGISEQ-2000 platform to generate 100-bp paired-end reads. Data were analyzed on http://biosys.bgi.com

### **SiRNA and shRNA lentivirus construction**

All siRNAs were purchased from Sangon Biotech, with sequences detailed in Supplementary Table I. Macrophages were transfected with siRNA or negative control siRNA using Lipofectamine 3000 (Invitrogen) or HiPerFect transfection reagent (Qiagen), according to the manufacturers' instructions.

shRNA lentiviral constructs were generated as previously described.^24^ Target sequences for short hairpin RNAs (shRNAs) were selected using BLOCK-iT™ RNAi Designer (Invitrogen) and/or i-Score Designer. shRNA constructs were generated using the pGreenPuro™ shRNA Cloning and Expression Lentivector Kit (System Biosciences Inc.), following the manufacturer's protocol. The non-targeting control shRNA was provided with the kit. For lentiviral particle packaging, shRNA lentivector was co-transfected with pMD2G and psPAX2 packaging plasmids into HEK293T cells.

### **ChIP-PCR**

Chromatin immunoprecipitation (ChIP) followed by PCR (ChIP-PCR) was conducted using the EZ-ChIP™ Chromatin Immunoprecipitation Kit (Millipore), following our previously reported methodology.^32^ Briefly, cells were crosslinked with 1% paraformaldehyde under rotation at room temperature for 10 minutes. Crosslinking was quenched by adding glycine to a final concentration of 0.125 M, followed by an additional 5 min of rotation. Cells were then washed three times with ice-cold PBS containing 1% PMSF and immediately resuspended in SDS lysis buffer supplemented with 1% PMSF. Lysates were sonicated on ice using a Bioruptor (Diagenode) for a total of 40 cycles (30 s on, 30 s off), performed in four increments of 10 cycles each. After pelleting cellular debris, the lysates were precleared by incubation with Protein G agarose for 1 h at 4 °C with rotation. For immunoprecipitation, precleared lysates were incubated overnight at 4 °C with rotation using the indicated antibodies. Protein G agarose was added for the final 2 h of incubation. The beads were subsequently washed sequentially with low-salt immune complex wash buffer, high-salt immune complex wash buffer, and LiCl immune complex wash buffer. Chromatin immunocomplexes were eluted by incubating the beads with elution buffer for 15  min at room temperature. To reverse the protein-DNA crosslinks, 5 M NaCl was added to the eluates, followed by overnight incubation at 65 °C. Following reversal of crosslinks, the samples were treated with RNase (30 min at 37 °C) and proteinase K (2  h at 55 °C). Finally, DNA was purified and subjected to ChIP-seq and qPCR analysis.

### **RNA extraction and qRT-PCR, Immunoblotting, H & E staining, immunostaining, and ELISA**

RNA extraction and qRT-PCR, immunoblotting, chromatin immunoprecipitation (ChIP)-polymerase chain reaction (PCR), Hematoxylin/eosin (H & E) staining, immunostaining, and enzyme linked immunosorbent assay (ELISA) were performed according to our previously reported methods.^24^^,^^25^^,^^29^^,^^33^

### **Statistical analyses**

Statistical analysis was performed using GraphPad Prism 8 software. Two side Student's t-test and ONE-way, ANOVA Bonferroni's Multiple Comparison Test were used to determine significance. Kaplan and Meier method was used to estimate the statistical significance of the survival curves. Mann-Whitney U test was used to analyze histological scores. A 95% confidence interval was considered significant and defined as *p* < 0.05. *indicates *p* < 0.05, **p < 0.01, ***p < 0.001. Ns, no significance.

## Results

### **Gut resident macrophages induced by butyrate-mediated LncLy6c exhibit high RUNX3 expression**

**Figure 1.**
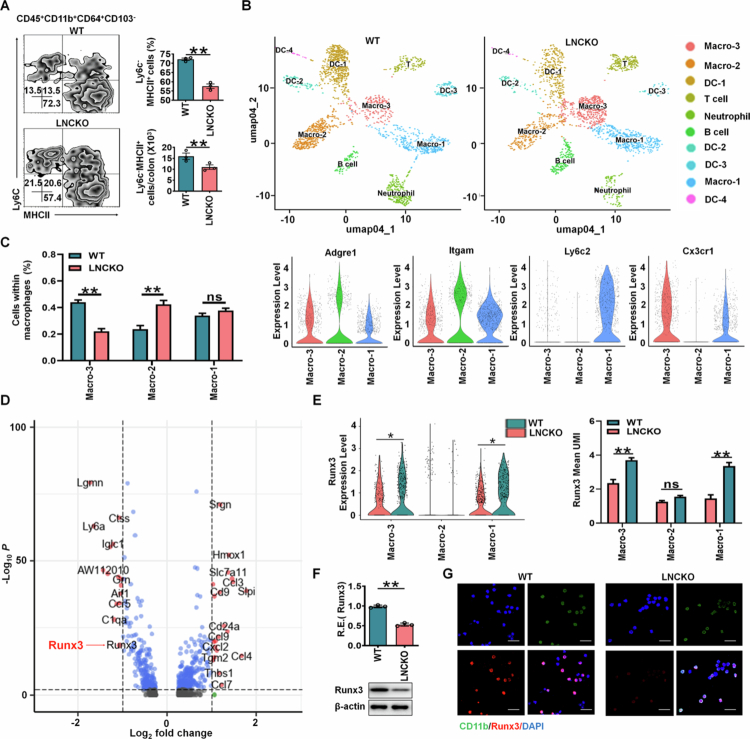
Gut resident macrophages induced by butyrate-mediated LncLy6c exhibit high RUNX3 expression. (A) Flow cytometry of CD45^+^CD11b^+^CD64^+^CD103^–^MHCII^+^Ly6C^–^ resident macrophages in the colon tissues of wt and *lncLy6c* KO (LNCKO) mice. (B) DESC clustering of CD11b^+^ cells in the colon tissues of control WT mice (*n* = 19895) and *lncLy6c* KO mice (LNCKO, *n* = 3245). Pooled sample from eight weeks-old male mice (*n* = 6). Macro, macrophages; DC, dendritic cells. (C) Proportions and characteristic markers of different macrophage subpopulations in total CD11b^+^ cells from the colon tissues of control WT and *lncLy6c* KO (LNCKO) mice. Macro-1,2 and 3, different macrophage subpopulations. (D) Volcano plot for visualizing different genes in colon macrophages of WT and *LncLy6c* KO (LNCKO) macrophages. Left, genes of high expression in the macrophages of control WT mice; Right, genes of high expression in the macrophages of *LncLy6c* KO (LNCKO) mice. (E) Violin plots and UMI showing expression levels of RUNX3 in the colon macrophage populations of WT and *lncLy6c* KO (LNCKO) macrophages. Macro-1,2 and 3, different macrophage subpopulations. (F) QRT-PCR (upon) and immunoblots (low) of RUNX3 in the colon resident macrophages in WT and *lncLy6c* KO (LNCKO) mice. R. E, relative expression. (G) Immunostaining of CD11b and RUNX3 in isolated colon resident macrophages of WT and *lncLy6c* KO (LNCKO) mice. Scale bar, 100 μM. ScRNA-seq data were obtained from 6 mice (C and E). The analyses in A and F are based on a sample size of *n* = 3 per group. Data are presented as the mean ± SD. Two side Student's t-test; *p < 0.05, **p < 0.01, ns, no significance; data were a representative of at least three experiments.

LncRNA *lncLy6c* which is directly regulated by butyrate,^24^ has been shown to promote the differentiation of peripheral blood Ly6C^high^ inflammatory macrophages into Ly6C^int/neg^ immunosuppressive macrophages.^24^ To further investigate this phenomenon in colon tissues, we compared *lncLy6c* knockout (KO) mice with wild-type (WT) controls. Flow cytometry analysis revealed a significant reduction in the proportion of gut resident macrophages (CD45^+^CD11b^+^CD64^+^CD103^–^MHCII^+^Ly6C^–/low^)^10-13^ in colon tissues of *lncLy6c* KO mice (Figure 1A). Single-cell RNA sequencing (scRNA-seq) analysis corroborated these findings, demonstrating a markedly decreased proportion of the resident macrophage population (Macro 3) in *lncLy6c* KO mice compared to WT controls (Figure 1B and C). These resident macrophages exhibited low expression of CCR2, F10, and Ly6C, along with high expression of immunosuppressive markers such as Mrc1 (CD206) and CX3CR1^10-13^ (Figure 1C, Supplementary Figure S1). Notably, no significant differences were observed in the proportion of CCR2^high^ F10^high^ Ly6C^high^ CX3CR1^low^ inflammatory monocytes/macrophages (Macro 1) between the two groups (Figure 1C, Supplementary Figure S1). Collectively, these data demonstrate that *lncLy6c* deficiency leads to a selective reduction in the resident macrophage population in colon tissues, without affecting inflammatory monocyte-derived macrophages.

To identify potential regulators of gut resident macrophage differentiation associated with *lncLy6c*, we analyzed single-cell RNA sequencing (scRNA-seq) data from *lncLy6c* KO and control mice. This analysis revealed multiple downregulated genes in KO resident macrophages, including the key transcription factor RUNX3 (Figure 1D and E). The reduction in RUNX3 expression was further validated at the transcriptional and protein levels using qRT-PCR, Western blotting, and immunostaining (Figure 1F and G). These findings demonstrate that butyrate-induced *lncLy6c* promotes the differentiation of gut resident macrophages with high RUNX3 expression.

### **Gut resident macrophages decreased in the colon tissue of *RUNX3*^*fl/fl-Lyz2-Cre*^ mice**

**Figure 2.**
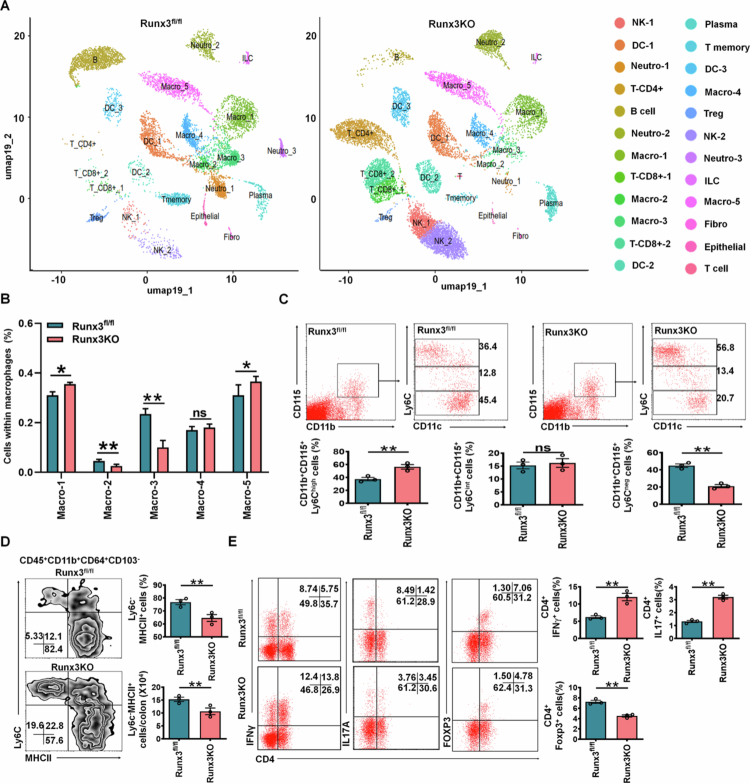
Gut resident macrophages decreased in the colon tissue of RUNX3^fl/fl-Lyz2-Cre^ mice. (A) DESC clustering of CD11b^+^ single cells in the colon tissues of *RUNX3*^*fl/fl-Lyz2-Cre*^ mice (RUNX3KO) and control RUNX3^fl/fl^ mice. Pooled sample from eight weeks-old male mice (*n* = 6). Macro, macrophages; DC, dendritic cells; Neutro, neutrophils. (B) Proportions of the populations of different macrophages in total CD11b^+^ cells from the colon tissues of *RUNX3*^*fl/fl-Lyz2-Cre*^ mice (RUNXKO) and control RUNX3^fl/fl^ mice. Macro-1,2,3,4 and 5, different macrophage subpopulations. (C) Flow cytometry of CD11b^+^CD115^+^Ly6C^high^, CD11b^+^CD115^+^Ly6C^int^, CD11b^+^CD115^+^Ly6C^neg^ cells in the bone marrow cells (BMCs) of *RUNX3*^*fl/fl-Lyz2-Cre*^ (RUNX3KO) and control RUNX3^fl/fl^ mice. (D) Flow cytometry of CD45^+^CD11b^+^CD64^+^CD103 ^–^MHCII^+^Ly6C^–^ resident macrophages in the colon tissues of *RUNX3*^*fl/fl-Lyz2-Cre*^ mice (RUNX3KO) and control RUNX3^fl/fl^ mice. (E) Flow cytometry of CD4^+^IFNγ^+^, CD4^+^IL-17A^+^and CD4^+^Foxp3^+^ T cells in the colon tissues of *RUNX3*^*fl/fl-Lyz2-Cre*^ (RUNX3KO) and control RUNX3^fl/fl^ mice. ScRNA-seq data were obtained from 6 mice (B). The analyses in C, D, and E are based on a sample size of *n* = 3 per group. Data are presented as mean ± SD. Two side Student's t-test; *p < 0.05, **p < 0.01; Ns, no significance. Data were a representative of at least three experiments.

RUNX3 has a potential role in maintaining intestinal homeostasis.^34^ This transcription factor contains an evolutionarily conserved DNA/protein-binding domain that serves as a key regulator of hematopoietic development and immune cell lineage commitment.^35-37^ Using a RUNX3 shRNA lentivirus mediated macrophage transplantation model, we found that *RUNX3* knockdown significantly reduced the number of resident macrophages (Supplementary Figure S2), indicating its essential role in promoting the differentiation of resident macrophages from inflammatory monocytes. To further investigate RUNX3's regulatory function in macrophage differentiation, we generated macrophage-specific *RUNX3* KO mice (*RUNX3*^*fl/fl-Lyz2-Cre*^) and compared them with *RUNX3*^*fl/fl*^ controls. scRNA-seq analysis of colonic macrophages revealed five distinct subpopulations (Figure 2A and B). Notably, the resident macrophage cluster (Macro−3), characterized by low CCR2 and F10 expression^38^ (Supplementary Figure 3, was significantly reduced in *RUNX3*^*fl/fl-Lyz2-Cre*^ mice, while CCR2^high^ F10^high^ inflammatory macrophage populations (Macro-5 and Macro-1)^39^ remained unchanged (Supplementary Figure S3). Flow cytometry analysis confirmed these findings, showing decreased CD11b^+^CD115^+^Ly6C^neg^ immunosuppressive macrophages in peripheral blood^14^^,^^24^ and reduced CD45^+^ CD11b^+^ CD64^+^ MHCII^+^ Ly6C^low/^^−^ resident macrophages^10-13^ in colonic tissues of *RUNX3*^*fl/fl-Lyz2-Cre*^ mice (Figure 2C and D). Complementary gene expression and signaling pathway analyses further validated these macrophage subpopulations (Supplementary Figure S4A and B), with pseudo-time analysis confirming that in cluster 3 (Macro-3) represents gut-resident macrophages while cluster 5 and 1 (Macro-5 and Macro-1) derive from inflammatory blood monocytes/macrophages (Supplementary Figure S4C). The depletion of these resident macrophages, known to contribute to inflammation,^17^^,^^18^ was accompanied by elevated Th1/Th17 responses and reduced Treg populations in colonic tissues (Figure 2E). Collectively, these results demonstrate that RUNX3 deficiency leads to decreased intestinal resident macrophages and concomitant increases in inflammatory cells within colonic tissue.

### ***RUNX3*^*fl/fl-Lyz2-Cre*^ mice are high sensitive to DSS mediated colitis**

**Figure 3.**
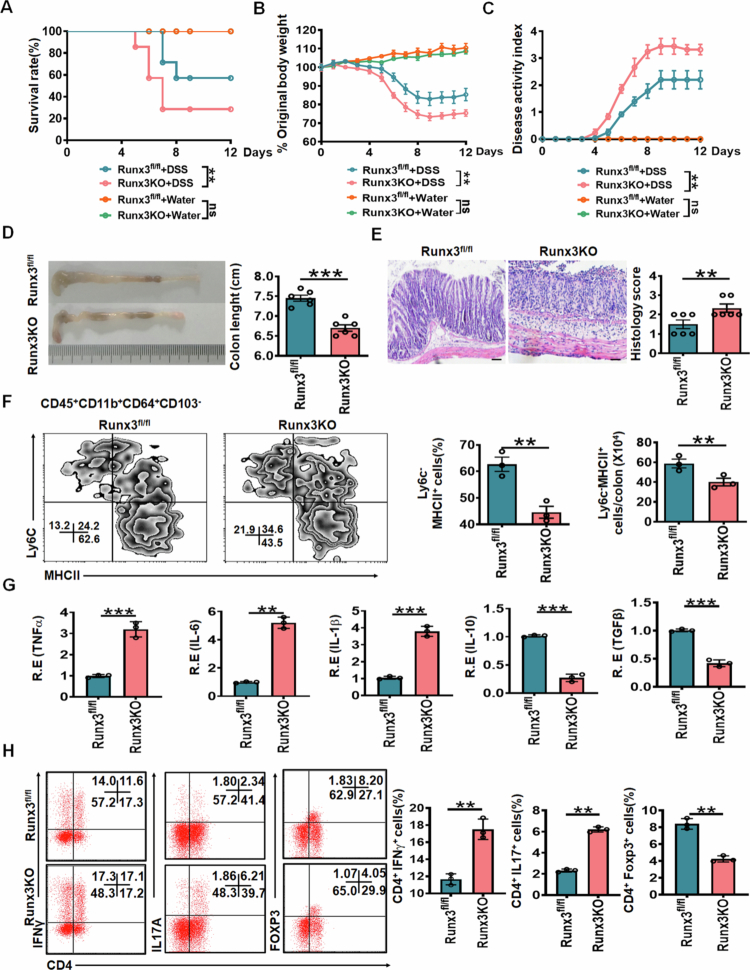
*RUNX3*^*fl/fl-Lyz2-Cre*^ mice are high sensitive to DSS-induced colitis. (A) Survival rate of *RUNX3*^*fl/fl-Lyz2-Cre*^ mice (RUNX3KO) and control RUNX3^fl/fl^ mice after DSS. (B) Body weight of *RUNX3*^*fl/fl-Lyz2-Cre*^ mice (RUNX3KO) and control RUNX3^fl/fl^ mice after DSS (*n* = 12). (C) Disease activity index of *RUNX3*^*fl/fl-Lyz2-Cre*^ mice (RUNX3KO) and control RUNX3^fl/fl^ mice after DSS. (D) Length of colon in *RUNX3*^*fl/fl-Lyz2-Cre*^ mice (RUNX3KO) and control RUNX3^fl/fl^ mice after DSS. (E) H&E staining and histology score of colon tissue of *RUNX3*^*fl/fl-Lyz2-Cre*^ mice (RUNX3KO) and control RUNX3^fl/fl^ mice after DSS. Scale bar, 40 μm. (F) Flow cytometry of CD45^+^CD11b^+^CD64^+^CD103^–^MHCII^+^Ly6C^–^ resident macrophages in the colon tissues of *RUNX3*^*fl/fl-Lyz2-Cre*^ mice (RUNX3KO) and control RUNX3^fl/fl^ mice after DSS. (G) qRT-PCR of TNFα, IL-6, IL-1β, IL-10 and TGFβ in the colon tissues of *RUNX3*^*fl/fl-Lyz2-Cre*^ mice (RUNX3KO) and control RUNX3^fl/fl^ mice after DSS.(H) Flow cytometry of CD4^+^IFN-γ^+^, CD4^+^IL17A^+^, CD4^+^Foxp3^+^ cells in the colon tissues of *RUNX3*^*fl/fl-Lyz2-Cre*^ mice (RUNX3KO) and control RUNX3^fl/fl^ mice after DSS. The analyses in A–C are based on a sample size of *n* = 12 per group, in D and E based on a sample size of *n* = 6 per group, and in F-H based on a sample size of *n* = 3 per group. Data are presented as mean ± SEM. Wilcoxon's test in A; One-way ANOVA test in B and C; Two side Student's t-test in other panels; *p < 0.05, **p < 0.01, ***p < 0.001.

To further investigate the role of RUNX3 in gut-resident macrophages, we utilized a DSS-induced colitis model. Compared to control *RUNX3*^*fl/fl*^ mice, *RUNX3*^*fl/fl-Lyz2-Cre*^ mice exhibited heightened susceptibility to chemically induced colitis, as evidenced by increased weight loss, reduced survival rates, more severe bleeding and diarrhea (higher disease index), shorter colon length, and elevated histological scores (Figure 3A–E). Additionally, colon tissues from *RUNX3*^*fl/fl-Lyz2-Cre*^ mice showed a significant reduction in resident macrophages (Figure 3F). Notably, *RUNX3*^*fl/fl-Lyz-Cre*^ mice also displayed elevated levels of pro-inflammatory cytokines (TNFα, IL-1β, and IL-6) alongside decreased anti-inflammatory cytokines (IL-10 and TGFβ) in colon tissues (Figure 3G). Given that these cytokines modulate the differentiation and function of Th1, Th17, and Treg cells,^40^^,^^41^ we further assessed these T-cell subsets and observed an increase in Th1 and Th17 cells but a decrease in Tregs in *RUNX3*^*fl/fl-Lyz2-Cre*^ mice (Figure 3H). These findings demonstrate that RUNX3-mediated regulation of gut-resident macrophages is essential for maintaining intestinal homeostasis.

### **RUNX3-mediated macrophages rely on Nr4A1-mediated ERK1/2/MAPK pathway**

**Figure 4.**
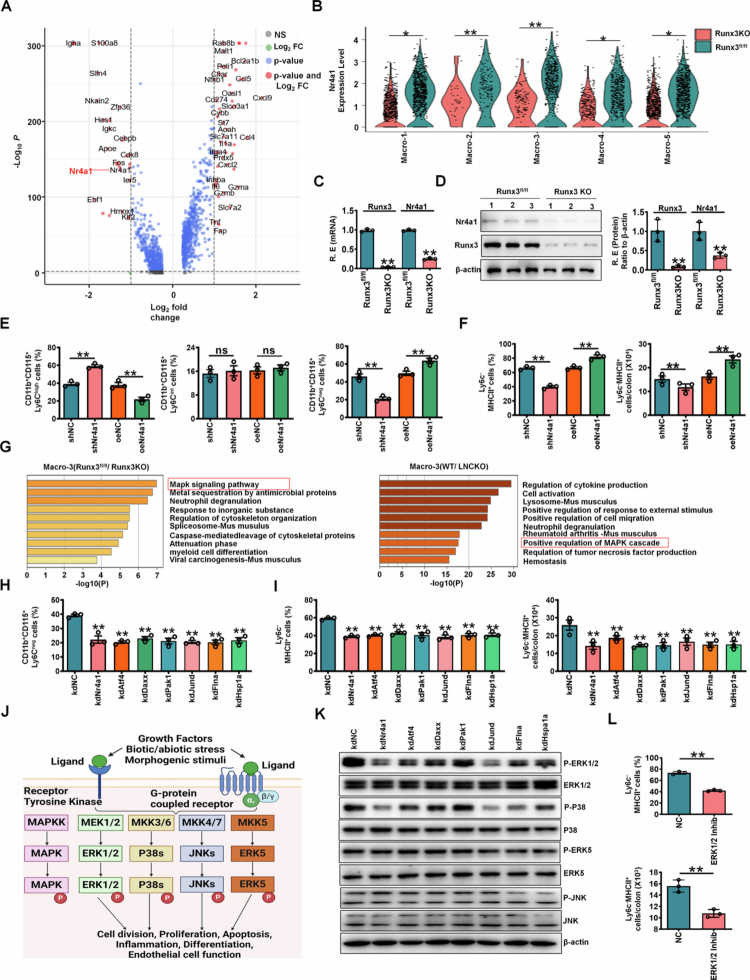
RUNX3-mediated macrophages rely on Nr4A1-mediated ERK1/2/MAPK pathway. (A) Volcano plot for visualizing genes of NR4A1 in the colon macrophages of *RUNX3*^*fl/fl-Lyz2-Cre*^ (RUNX3KO) and control RUNX3^fl/fl^ mice. Left, genes of high expression in the macrophages of control RUNX3^fl/fl^ mice; Right, genes of high expression in the macrophages of *RUNX3*^*fl/fl-Lyz2-Cre*^ mice. (B) Violin plots showing different expression levels of NR4A1 in the colon macrophages across different clusters. Macro-1, 2, 3, 4 and 5, different macrophage subpopulations. (C and D) QRT-PCR (left) and Western blot (right) of RUNX3 and NR4A1 in the colon resident macrophages of *RUNX3*^*fl/fl-Lyz2-Cre*^ (RUNX3KO) and control RUNX3^fl/fl^ mice. The macrophages were isolated from the colon tissue of mice. R. E, relative expression. (E) Flow cytometry of CD11b^+^CD115^+^Ly6C^high^, CD11b^+^CD115^+^Ly6C^int^, and CD11b^+^CD115^+^Ly6C^neg^ cells in the BMCs of Nr4a1 shRNA (shNr4a1) or Nr4A1 (oeNr4a1) lentivirus infected macrophages transplantation mice. shNC, control shRNA lentiviruses; oeNC, control lentiviruses. (F) Flow cytometry of CD45.1^+^CD11b^+^CD64^+^CD103^–^MHCII^+^Ly6C^–^ resident macrophages in colon tissues of Nr4a1 shRNA (shNr4a1) or Nr4a1 (oeNr4a1) lentiviruses infected macrophages transplantation model. shNC, control shRNA lentiviruses; oeNC, control lentiviruses. (G) KEGG and GSEA analyses of the colon resident macrophage from *RUNX3*^*fl/fl-Lyz2-Cre*^ (RUNX3KO) and control RUNX3^fl/fl^ mice (left), or from WT and *lncLy6c* KO (LNCKO) mice (right). (H) Flow cytometry of CD11b^+^CD115^+^Ly6C^high^, CD11b^+^CD115^+^Ly6C^int^, CD11b^+^CD115^+^Ly6C^neg^ cells in the BMCs of different shRNA infected macrophage transplantation mice. KdNC, control shRNA lentiviruses; Kd, knockdown. (I) Flow cytometry of CD45.1^+^CD11b^+^CD64^+^CD103 ^–^ MHCII^+^Ly6C^–^ resident macrophages in colon tissues of different shRNA lentiviruses infected macrophage transplantation model. KdNC, control shRNA lentiviruses; Kd, knockdown. (J) A schematic illustration showing different cascades in MAPK pathways. (K) Western blot of P (phosphorylated)-ERK1/2, ERK1/2, P-p38, P38, P-ERK5, ERK5, P-JNK and JNK in different shRNA infected mouse macrophages from bone marrow cells. Kd, knockdown. (L) Flow cytometry of CD45.1^+^CD11b^+^CD64^+^CD103 ^–^MHCII^+^Ly6C^–^ resident macrophages in colon tissues of ERK1/2 inhibitor treated mice. In E–F, shNC (Macrophages infected with control shRNA) lentiviruses), shNr4a1 (#acrophages infected with shNr4a1 lentiviruses), oeNC (macrophages infected with control lentiviruses) and oeNr4a1 (macrophages infected with Nr4a1 lentiviruses) were transplanted into WT mice. In H and I, macrophages infected with different shRNA (kdNr4a1, kdAtf4, kdDaxx, kdPak1, kdJund, kdFlna and kdHSP1a) lentiviruses were transplanted into WT mice. ScRNA-seq data were obtained from 6 mice (B). The analyses in C–F, H, I and L are based on a sample size of *n* = 3 per group. Data are presented as the mean ± SD. Two side Student's t-test; *p < 0.05, **p < 0.01; Ns, no significance. Data were a representative of at least three experiments.

To identify the key factor mediating RUNX3-dependent macrophage differentiation, we focused on Nr4a1, a known regulator of resident macrophage development.^14^ Single-cell RNA sequencing (scRNA-seq) revealed significantly reduced Nr4a1 expression in colon macrophages from *RUNX3*^*fl/fl-Lyz2-Cre*^ mice compared to controls (Figure 4A). Intriguingly, this downregulation was most pronounced in the Macro-3 subset (resident macrophages), while other macrophage populations (Macro-1, -4, and -5) showed only modest reductions (Figure 4B). Consistent with these findings, qRT-PCR and Western blot also confirmed lower Nr4a1 levels in isolated RUNX3-deficient resident macrophages (Figure 4C and D). Notably, in the Nr4a1 shRNA lentivirus-infected macrophage transplantation model, we observed a significant reduction in immunosuppressive CD11b^+^CD115^+^Ly6C^neg^ resident macrophages in peripheral blood^14^^,^^24^ as well as decreased CD45^+^CD11b^+^CD64^+^MHCII^+^Ly6C^low/^^−^ resident macrophages^10-13^ in colon tissues. In contrast, mice receiving Nr4a1 lentivirus-infected macrophages exhibited not only a marked increase in peripheral CD11b^+^CD115^+^Ly6C^neg^ resident macrophages but also elevated levels of CD45^+^CD11b^+^CD64^+^MHCII^+^Ly6C^low/^^−^ resident macrophages in colon tissues (Figure 4E and F; Figure S5A). These findings further support the critical role of Nr4a1 in promoting the differentiation of gut-resident macrophages. Taken together, our results demonstrate that RUNX3-associated Nr4a1 can effectively induce the development of gut-resident macrophages.

To further elucidate the mechanisms by which Nr4a1 induces the differentiation of resident macrophages, we performed KEGG and GSEA analyses. These analyses revealed a distinct enrichment of the mitogen-activated protein kinase (MAPK) signaling pathway in resident macrophages (Macro-3) compared to other macrophage subsets (Figure 4G). Notably, this MAPK pathway signature was also observed in the resident macrophage population (Macro−3) identified in Figure 1 (Figure 4G), suggesting a consistent association between MAPK signaling and resident macrophage identity. Collectively, these findings indicate that Nr4a1 may promote resident macrophage differentiation through a unique MAPK-dependent mechanism. Comparative analysis of scRNA-seq data from *LncLy6c* KO and *RUNX3*^*fl/fl-Lyz2-iCre*^ mice revealed that the MAPK signaling pathway in resident macrophages involves not only Nr4a1 but also key regulators such as *Atf4*, *Daxx*, *Jund*, *Pak1*, *Flna*, and *Hspa1a*. To assess their functional roles, we systematically evaluated the impact of these genes on resident macrophage differentiation. Strikingly, each gene significantly influenced the differentiation process (Figure 4H and I; Figure S5B), further supporting the critical involvement of MAPK signaling in shaping the resident macrophage phenotype. Notably, the MAPK pathway comprises four major subfamilies: extracellular signal-regulated kinases (ERK1/2), c-Jun *N*-terminal kinases (JNK1/2/3), p38-MAPK, and ERK5^42^ (Figure 4J). This hierarchical organization suggests potential mechanistic diversity in how MAPK signaling orchestrates macrophage differentiation. To delineate the functional contributions of Nr4A1, Atf4, Daxx, Jund, Pak1, Flna, and Hspa1a to the four MAPK subfamilies, we performed targeted gene silencing experiments. Strikingly, knockdown of any of these genes significantly attenuated ERK1/2 MAPK activation, while leaving ERK5 and JNK pathways unaffected (Figure 4K). Notably, since Nr4A1, Atf4, Daxx, Jund, Pak1, Flna, and Hspa1a all significantly influenced the differentiation process, only Nr4A1, Jund and Flna were found to affect p38 activation (Figure 4K), suggesting that the p38-MAPK pathway is not involved in RUNX3-mediated macrophage differentiation**.** This selective impairment strongly implicates the ERK1/2/MAPK pathway as the primary mediator of RUNX3-dependent resident macrophage differentiation. Consistent with this mechanism, pharmacological inhibition of ERK1/2/MAPK signaling in vivo robustly suppressed resident macrophage differentiation (Figure 4L). Taken together, these results establish that RUNX3 promotes resident macrophage development through an Nr4a1-dependent ERK1/2/MAPK signaling axis.

### **RUNX3 promotes expression of ZFP36 through Nr4A1/ERK1/2/MAPK pathway.**

**Figure 5.**
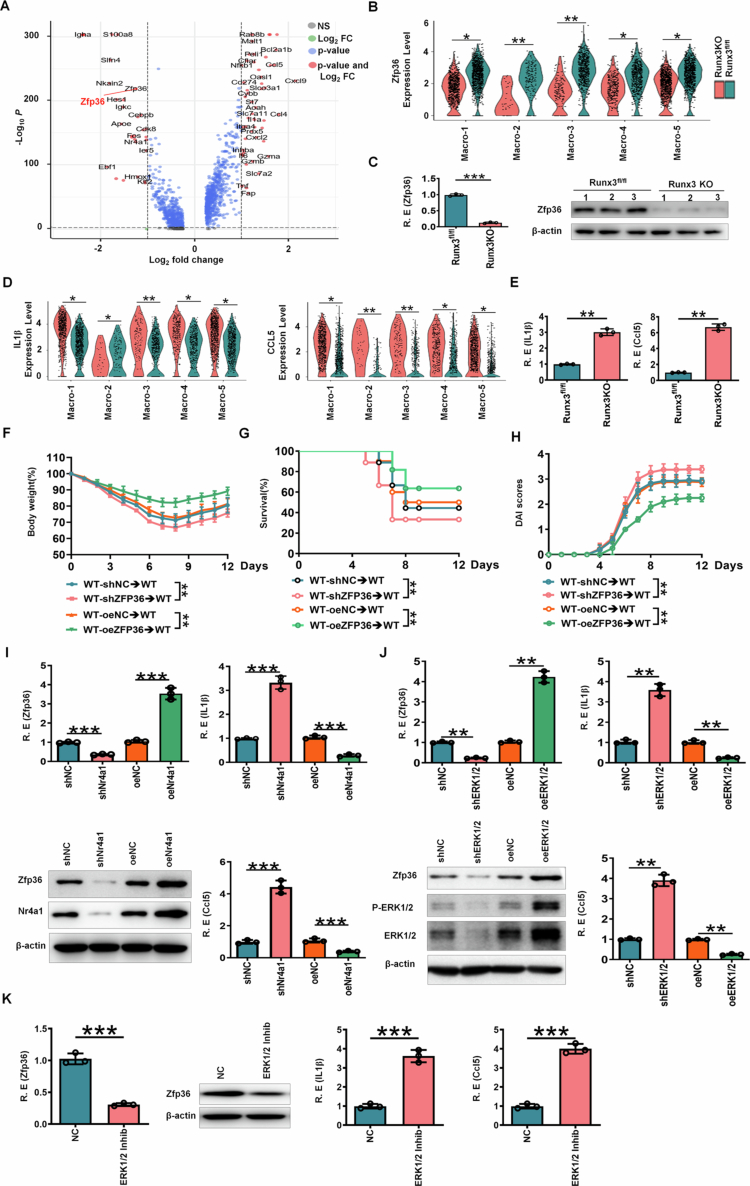
RUNX3 promotes expression of ZFP36 through Nr4A1/ERK1/2/MAPK pathway. (A) Volcano plot for visualizing genes of ZFP36 in the colon resident macrophages of *RUNX3*^*fl/fl-Lyz2-Cre*^ mice (RUNX3KO) and control RUNX3^fl/fl^ mice. Left, genes of high expression in the macrophages of control mice; Right, genes of high expression in the macrophages of *RUNX3*^*fl/fl-Lyz2-Cre*^ mice. (B) Violin plots of ZFP36 in different macrophage populations of *RUNX3*^*fl/fl-Lyz2-Cre*^ (RUNX3KO) and control RUNX3^fl/fl^ mice. Macro-1, 2, 3, 4 and 5, different macrophage subpopulations. (C) QRT-PCR (left) and Western blot (right) of ZFP36 in the colon resident macrophages of *RUNX3*^*fl/fl-Lyz2-Cre*^ (RUNX3KO) and control RUNX3^fl/fl^ mice. The macrophages were isolated from the colon tissues of mice. R. E, relative expression. (D) Violin plots of IL-1β and CCL5 in different macrophage populations of *RUNX3*^*fl/fl-Lyz2-Cre*^ (RUNX3KO) and control RUNX3^fl/fl^ mice. Macro-1, 2, 3, 4 and 5, different macrophage subpopulations. (E) QRT-PCR of IL-1β and CCL5 in the colon resident macrophages of *RUNX3*^*fl/fl-Lyz2-Cre*^ mice (RUNX3KO) and control RUNX3^fl/fl^ mice. R. E, relative expression. (F) Weight changes in macrophages transplanted mice after DSS. (G) Mortality rate in macrophages transplanted mice after DSS. (H) Disease activity index (DAI) in macrophages transplanted mice after DSS. (I) QRT-PCR of ZFP36, IL-1β and CCL5, and immunoblotting of ZFP36 and Nr4A1 in shRNA Nr4a1 infected resident macrophages or Nr4a1 lentiviruses infected resident macrophages. shNC, shRNA negative control; shNr4a1, Nr4a1 shRNA; oeNC, Nr4a1 control; oeNr4a1, exogenous Nr4a1. (J) QRT-PCR of ERK1/2, ZFP36, IL-1β and CCL5, and immunoblotting of ZFP36 in ERK1/2 silencing (shRNA) or overexpressing (oe) lentiviruses infected resident macrophages. shNC, shRNA control; shERK1/2, ERK1/2 shRNA; oeNC, ERK1/2 control; oeERK1/2, exogenous ERK1/2. (K) QRT-PCR of ZFP36, IL-1β and CCL5, and immunoblotting of ZFP36 in ERK1/2 inhibitor (Inhib) treated colon resident macrophages (100 mM, MCE, USA). NC, negative control. In F–H, WT-shNC→WT (WT mice derived macrophages infected with control shRNA (shNC) lentiviruses), WT-shZFP36→WT (WT mice derived macrophages infected with shZFP36 lentiviruses), WT-oeNC→WT (WT mice derived macrophages infected with control lentiviruses (oeNC)) and WT-oeZFP36→WT (WT mice derived macrophages infected with ZFP36 lentiviruses (oeZFP36)) were transplanted into WT mice. ScRNA-seq data were obtained from 6 mice (B and D); The analyses in C, E, I–K are based on a sample size of *n* = 3 per group; The analyses in F, G and H are based on a sample size of *n* = 12 per group; data are presented as the mean ± SD. Wilcoxon's test in G; One-way ANOVA test in F and H; Two side Student's t-test in other panels; *p < 0.05, **p < 0.01, ***p < 0.001. Data were a representative of at least three experiments.

Macrophages mediate their biological functions primarily through cytokine and chemokine secretion.^43^ Recent studies have revealed that RUNX3-deficient macrophages display a distinct immunoregulatory imbalance, marked by both reduced expression of anti-inflammatory mediators and defective suppression of pro-inflammatory genes.^37^ Since ZFP36 (zinc finger protein 36) promotes the degradation of cytokine and chemokine mRNAs containing AU-rich elements (AREs) in their 3'-UTRs,^44^^,^^45^ we next examined ZFP36 expression in macrophages. Our data revealed a significant reduction in ZFP36 levels in *RUNX3* KO colon-resident macrophages compared to *RUNX3*^*fl/fl*^ controls (Figure 5A). Single-cell RNA sequencing analysis further demonstrated that ZFP36 expression was markedly decreased in resident macrophages (Macro 3 cluster) from *RUNX3* KO mice relative to control mice (Figure 5B). Consistent with these findings, qRT-PCR and immunoblotting analyses confirmed the downregulation of ZFP36 in colon-resident macrophages from *RUNX3*^*fl/fl-Lyz2-Cre*^ mice (Figure 5C). scRNA- sequencing analysis demonstrated more pronounced reductions in IL-1β and CCL5 expression within resident macrophages (Macro 3) of *RUNX3* KO mice relative to controls (Figure 5D). qRT-PCR analysis confirmed the reduction of key cytokines and chemokines, including IL-1β and CCL5 (Figure 5E). Notably, mice receiving ZFP36 shRNA lentivirus-infected macrophages exhibited heightened susceptibility to chemically induced colitis compared to controls, as evidenced by more pronounced weight loss, reduced survival rates, increased bleeding and diarrhea severity, and higher overall disease activity indices (Figure 5F–H; Supplementary Figure S5C). In contrast, transplantation of ZFP36-overexpressing macrophages conferred significant protection against DSS-induced colitis. These findings underscore the critical role of ZFP36 in resident macrophages for maintaining intestinal homeostasis.

The expression of ZFP36 is known to be regulated by the ERK signaling pathway.^46^ Given the involvement of Nr4a1/ERK1/2 MAPK signaling in RUNX3-mediated regulation of resident macrophages, we further investigated its role in modulating ZFP36 expression in these cells. Silencing Nr4a1 or ERK1/2 not only downregulated ZFP36 expression but also enhanced the production of pro-inflammatory cytokines, including IL-1β and CCL5. Conversely, Nr4a1 or ERK1/2 overexpression upregulated ZFP36 while suppressing IL-1β and CCL5 expression (Figure 5I and J). Similarly, inhibition of ERK1/2 signaling also reduced ZFP36 levels and increased IL-1β and CCL5 expression (Figure 5K). Additionally, we examined the interplay between resident macrophage-derived chemokines and lymphocytes, observing enhanced chemokine-lymphocyte interactions (Supplementary Figure S6A). These interactions may contribute to the elevated frequencies of Th1 and Th17 cells, along with reduced Treg cell populations, in the colon tissues of *RUNX3*^*fl/fl-Lyz2-Cre*^ mice (Supplementary Figure S6B).^40^^,^^41^ Collectively, these findings demonstrate that RUNX3 enhances ZFP36 expression and attenuates pro-inflammatory cytokine and chemokine production in colon macrophages via the Nr4a1-associated ERK1/2/MAPK pathway.

### **Butyrate promotes RUNX3 expression through lncLy6c mediated H3K4me3 modification**

**Figure 6.**
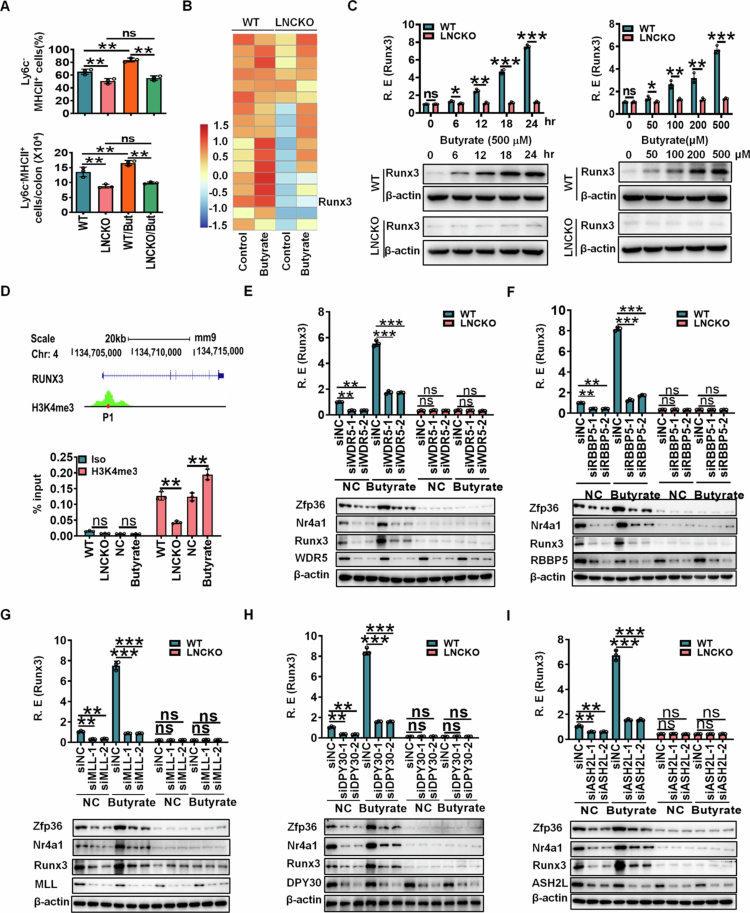
Butyrate promotes RUNX3 expression through lncLy6c mediated H3K4me3 modification. (A) Flow cytometry of CD45^+^CD11b^+^CD64^+^CD103^–^MHCII^+^Ly6C^–^ resident macrophages in colon tissues of *lncLy6c* KO (LNCKO) and control wt mice with (WT/But and LNCKO/But) or without (WT and LNCKO) butyrate. (B) RNA-Seq of *lncLy6c* KO (LNCKO) and control wt macrophages upon exposure to butyrate for 24 h. Macrophages were generated from bone marrow cells. (C) QRT-PCR (above) and western blot (below) of RUNX3 in *lncLy6c* KO (LNCKO) and control WT macrophages upon exposure to butyrate at different times and different concentration. Macrophages were generated from bone marrow cells. WT, the macrophages from WT mice; LNCKO, the macrophages from *lncLy6c* KO mice. (D) A schematic illustration (above) showing enrichment of H3K4me3 on the promoter region of RUNX3, and ChIP-PCR (below) of H3K4me3 marks on the promoter region of RUNX3. % input was compared. WT, only WT macrophages; LNCKO, only *lncLy6c* KO macrophages; NC, WT macrophages + control; Butyrate, WT macrophages + butyrate. (E–I) QRT-PCR (Above) of RUNX3 and Western blot (lower) of RUNX3, ZFP36, NR4A1 in the BMDMs from *lncLy6c* KO (LNCKO) and control WT mice after silencing WDR5 (siWDR5, E), RBBP5 (siRBBP5, F), MLL (siMLL, G), DPY30 (siDPY30, H) and ASH2L (siASH2L, I) upon exposure to butyrate. Si-NC, siRNA control; NC, butyrate control. The analyses in A, C–I are based on a sample size of *n* = 3 per group. Data are presented as the mean ± SD. Two side Student's t-test; *p < 0.05, **p < 0.01, ***p < 0.001.

We have previously demonstrated that the gut microbiota metabolite butyrate promotes the differentiation of Ly6C^low^ immunosuppressive macrophages through lncLy6c.^24^ In this study, we further confirmed that butyrate enhances the differentiation of Ly6C^low^ gut-resident macrophages in the colonic tissues of wild-type (wt) mice, but not in *lncLy6c*-deficient mice^24^ (Figure 6A). Given that butyrate-mediated lncLy6c regulates RUNX3 expression (Figure 1D), we hypothesized that butyrate might also modulate RUNX3 levels. Consistent with this notion, RNA sequencing revealed elevated RUNX3 expression in macrophages from wt mice, but not in those from lncLy6c-deficient mice following butyrate treatment (Figure 6B). These findings were corroborated by qRT-PCR and immunoblot analyses (Figure 6C). Previous studies have shown that butyrate-mediated lncLy6c can interact with various H3K4me3-associated proteins to enhance gene expression.^24^^,^^47^ Genome browser analysis further demonstrated H3K4me3 enrichment at the RUNX3 promoter region (Figure 6D). We therefore investigated whether lncLy6c influences H3K4me3 enrichment at the RUNX3 promoter to regulate its expression. Chromatin immunoprecipitation (ChIP)-PCR assays indeed showed that lncLy6c-deficient macrophages exhibited reduced H3K4me3 enrichment at the RUNX3 promoter (Figure 6D). Conversely, butyrate treatment increased H3K4me3 marks at this region (Figure 6D). Furthermore, knockdown of H3K4me3-related proteins - including WDR5 (WD repeat-containing protein 5), RBBP5 (retinoblastoma-binding protein 5), MLL (mixed lineage leukemia), DPY30, and ASH2L (absent-small-homeotic−2-like protein) – significantly affected RUNX3 expression (Figure 6E–I). These results suggest that butyrate-mediated lncLy6c upregulates RUNX3 expression through H3K4me3 modification. Given the established relationship between RUNX3 and the expression of Nr4a1 and ZFP36 in resident macrophages (Figures 4 and 5), we also examined these factors. Silencing of WDR5, ASH2L, MLL, RBBP5, or DPY30 similarly altered Nr4a1 and ZFP36 expression following butyrate exposure (Figure 6E–I). Collectively, these findings demonstrate that butyrate induces RUNX3 expression via lncLy6c-mediated H3K4me3 modification.

### **Butyrate promotes differentiation of gut resident macrophages through RUNX3**

**Figure 7.**
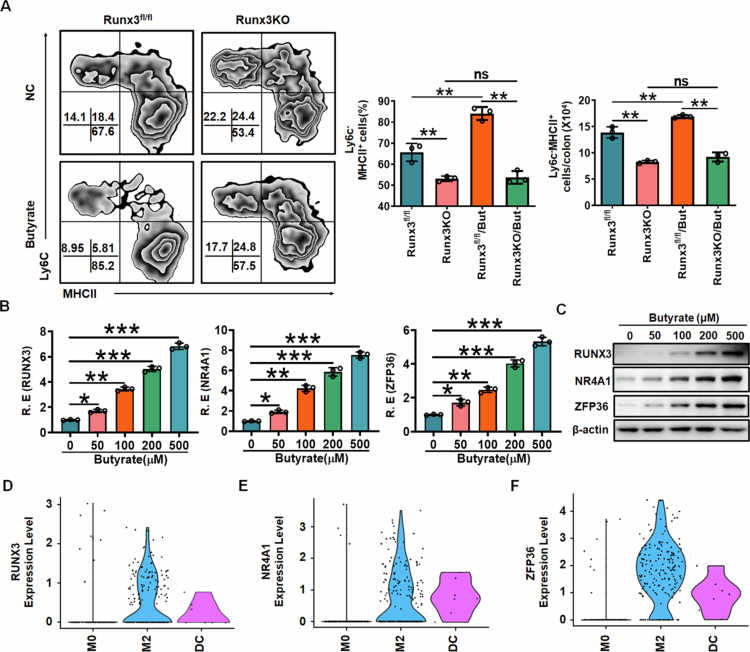
Butyrate promotes differentiation of gut resident macrophages through RUNX3. Flow cytometry of CD45^+^CD11b^+^CD64^+^CD103 ^–^MHCII^+^Ly6C^–^ resident macrophages in the colon tissues of *RUNX3*^*fl/fl-Lyz2-Cre*^ mice (RUNX3KO) and RUNX3^fl/fl^ mice with or without butyrate treatment (*n* = 3). (B) QRT-PCR of RUNX3, NR4A1 and ZFP36 in human monocytes derived macrophages after exposure to different concentrations of butyrate. (C) Immunoblots of RUNX3, NR4A1 and ZFP36 in human monocytes derived macrophages after exposure to different concentrations of butyrate. (D–F) Violin plots of RUNX3, NR4A1 and ZFP36 in different macrophage populations of healthy individuals. M0, macrophage 0; M2, macrophage 2 (resident macrophage); DC, dendritic cells. The analyses in A and B are based on a sample size of *n* = 3 per group. Data are presented as the mean ± SD. Two side Student's t-test; *p < 0.05, **p < 0.01, ***p < 0.001. Data were a representative of at least three experiments.

We finally investigated the effects of butyrate on the differentiation of colon-resident macrophages. After administering sodium butyrate (NaB) to mice, we observed that butyrate effectively promoted the differentiation of colon-resident macrophages in *RUNX3*^*fl/fl*^ mice but had no such effect in *RUNX3*^*fl/fl-Lyz2-Cre*^ mice (Figure 7A).

Notably, healthy individuals typically exhibit higher levels of short-chain fatty acids (SCFAs) in their stool and serum compared to patients with inflammatory bowel diseases (IBD), such as ulcerative colitis (UC) and Crohn's disease (CD).^48^ Given that the SCFA butyrate can induce RUNX3 expression, these findings suggest that elevated SCFA levels in healthy individuals may also contribute to the expression of RUNX3, Nr4a1, and ZFP36 in gut-resident macrophages. Consistent with this, in vitro experiments confirmed that butyrate upregulates the expression of RUNX3, Nr4a1, and ZFP36 in human macrophages (Figure 7B and C). We further examined the expression of RUNX3, Nr4a1, and ZFP36 in colon-resident macrophages from healthy individuals. According to the findings of Garrido-Trigo et al.,^49^ macrophages in UC and CD patients could be classified into five subsets: M0, M2 (resident) macrophages, two transcriptionally distinct M1 populations (M1 ACOD1 and M1 CXCL5), and inflammation-dependent alternative (IDA) macrophages. However, healthy individuals exhibited only two subsets: M0 and resident (M2) macrophages.^49^ Notably, resident macrophages (M2) in healthy individuals displayed significantly higher expression levels of Nr4a1, ZFP36, and RUNX3 (Figure 7D–F). Intriguingly, in the colon tissues of IBD patients, RUNX3, Nr4a1, and ZFP36 were highly expressed not only in resident macrophages but also in inflammatory macrophages (Supplementary Figure S7), indicating that their expression in macrophages may also be influenced by the inflammatory microenvironment. Thus, there exists elevated expression of RUNX3, Nr4a1, and ZFP36 in colon-resident macrophages from healthy individuals.

## Discussion

**Figure 8.**
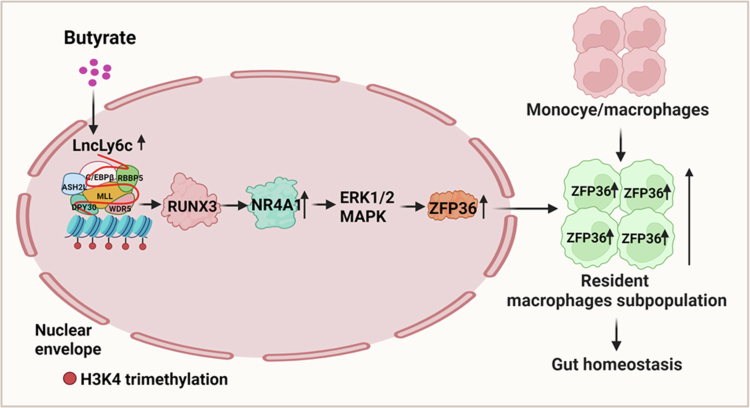
Graphical abstract. RUNX3 can induce Nr4a1^high^ZFP36^high^ resident macrophages to maintain gut homeostasis. Nr4a1/ERK1/2/MAPK pathway is involved in the differentiation of RUNX3 mediated gut resident macrophages and ZFP36 expression. RUNX3 expression in the colon resident macrophages can be induced by butyrate via *lncLy6c*. Thus, butyrate-mediated RUNX3 promotes differentiation of Nr4a1^high^ZFP36^high^ resident macrophages via NR4A1/ERK1/2 MAPK to maintain gut homeostasis.

Here, we demonstrate that gut microbiota-derived butyrate promotes intestinal homeostasis by enhancing the differentiation of Nr4a1^high^ZFP36^high^ macrophages through RUNX3 activation. Our findings reveal that Nr4A1 upregulation in gut-resident macrophages mediates the ERK1/2/MAPK pathway, which plays a crucial role in RUNX3-dependent macrophage differentiation and ZFP36 expression. The gut microbiota metabolite butyrate can induce RUNX3 expression in colonic resident macrophages via LncRNA lncLy6c-mediated H3K4me3 modification. Consistent with this mechanism, healthy individuals exhibit significantly higher fecal and serum butyrate levels compared to IBD patients.^48^ Correspondingly, colonic resident macrophages from healthy donors show elevated expression of RUNX3, Nr4a1, and ZFP36. In summary, our study establishes a butyrate-RUNX3-Nr4A1/ERK1/2 MAPK-ZFP36 axis that drives the differentiation of specialized resident macrophages to maintain intestinal homeostasis (Figure 8).

Our findings demonstrate that RUNX3 mediates the generation of Nr4a1^high^ZFP36^high^ resident macrophages, with ZFP36 playing a critical role in maintaining gut homeostasis. The importance of RUNX3 in intestinal health is well-established. RUNX3 deficiency has been linked to the development of IBD, including UC and CD.^50^ Genetic variants in the RUNX3 locus have been consistently associated with both CD and UC,^51^ and reduced RUNX3 expression has been shown to promote UC-associated tumorigenesis.^52^ Genome-wide association studies (GWAS) have further identified RUNX3 locus variants associated with celiac disease and UC,^51^ while additional GWAS data reveal associations between RUNX3 susceptibility loci and various inflammatory disorders, including CD (https://www.ebi.ac.uk/gwas/genes/RUNX3).^53^ ZFP36 can serve as a crucial regulator of inflammatory responses.^54^ This RNA-binding protein promotes the degradation of multiple cytokine and chemokine mRNAs.^44^ ZFP36 deficiency in knockout mice leads to accumulation of pro-inflammatory cytokines^55^ and results in severe systemic inflammatory syndrome,^56^ underscoring its essential role in inflammation control.

We also demonstrate that the RUNX3-mediated induction of Nr4a1^high^ZFP36^high^ resident macrophages is dependent on the ERK1/2 MAPK pathway. Previous studies have established that Nr4a1 is highly expressed in patrolling monocytes and is essential for their survival.^57^ Importantly, Nr4a1 plays a critical role in the differentiation of inflammatory monocytes into resident macrophages in the gut,^14^^,^^15^ with *Nr4a1*^−/−^ mice failing to develop adequate macrophage populations.^58^ The ERK1/2 pathway is known to be crucial for macrophage differentiation and function, regulating cytokine production at both transcriptional and post-transcriptional levels.^59^ Notably, inhibition of ERK phosphorylation has been shown to block M2 macrophage reprogramming.^60^ Furthermore, ERK1/2 phosphorylates downstream kinases such as MSKs, which also contribute to M2 macrophage differentiation.^61^ Interestingly, while the MAPK pathway has been implicated in inflammatory macrophage function,^62^^,^^63^ our findings suggest that distinct MAPK signaling cascades may underlie these differential effects.

The gut microbiota-derived metabolite butyrate has been shown to upregulate RUNX3 expression, thereby enhancing the differentiation of colon-resident macrophages. Accumulating evidence suggests that butyrate exerts immunomodulatory effects on macrophages through multiple mechanisms. Notably, butyrate can suppress LPS-induced production of proinflammatory mediators in macrophages.^64^ Furthermore, sodium butyrate (NaB) promotes macrophage polarization toward the M2 phenotype via acetylation-dependent pathways, including STAT1 and NF-κB subunit p65 acetylation.^65^ This M2-polarizing effect has therapeutic implications, as adoptive transfer of butyrate-induced M2 macrophages has been demonstrated to facilitate mucus layer restoration following dextran sulfate sodium (DSS)-induced colonic injury.^66^ Interestingly, contrasting reports indicate that butyrate may also function as a microbiota-derived danger signal, capable of regulating NLRP3 inflammasome activation through epigenetic modulation of inflammatory responses in human macrophages.^67^

## Supplementary Material

Supplementary material

## Data Availability

GEO accession number: https://www.ncbi.nlm.nih.gov/geo/query/acc.cgi?acc=GSE287023; Single cell mRNA sequencing: https://www.ncbi.nlm.nih.gov/geo/query/acc.cgi?acc=GSE287970, https://www.ncbi.nlm.nih.gov/geo/query/acc.cgi?acc=GSE288190; The data that support the findings of this study are available on reasonablerequest from the corresponding author.
