# Supplementary material for: Gut microbiota butyrate mediated RUNX3 promotes Nr4a1highZFP36high resident macrophages via NR4A1/ERK1/2 MAPK to maintain gut homeostasis
Source: Gut Microbes. 2025 Oct 17;17(1):2569741. doi: 10.1080/19490976.2025.2569741 (PMC12536639; doi:10.1080/19490976.2025.2569741)
Supplement: Supplementary material [file KGMI_A_2569741_SM7609.docx]

**Gut microbiota butyrate mediated RUNX3 promotes Nr4a1^high^ZFP36^high^ resident macrophages via NR4A1/ERK1/2 MAPK to maintain gut homeostasis**

Yunhuan Gao^1*^, Yi Shi^2*^, Ningning Zhu^1^, Yang Hao^1^, Juanjuan Wang^1^, Yuan Zhang^1^, and Rongcun Yang^1,3,4^


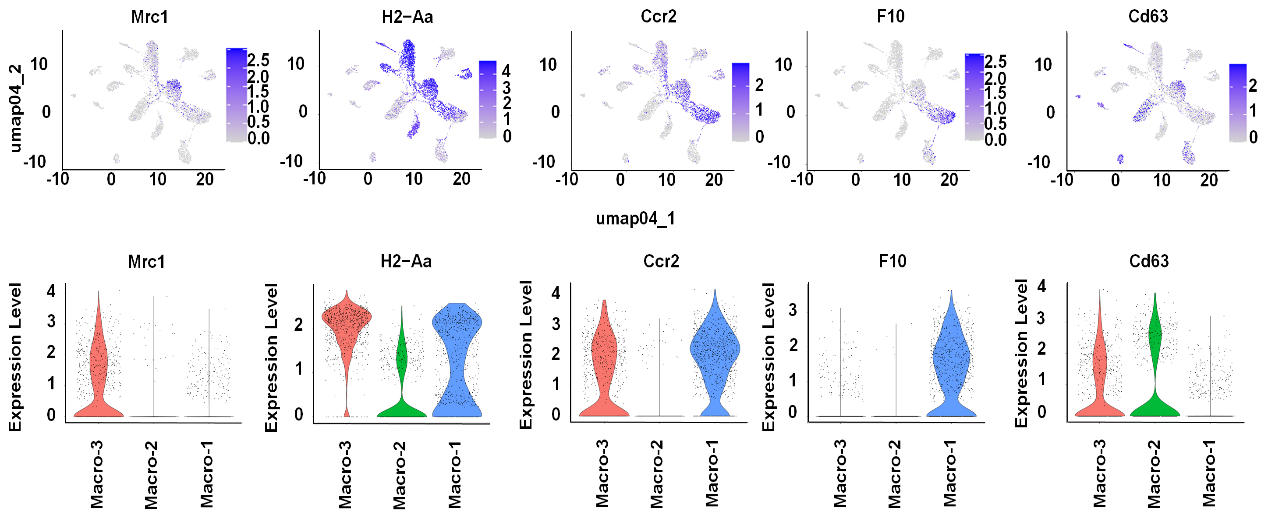


**Figure S1. Feature plots and violin plots in different macrophage subpopulations.** Feature plots and violin plots showing different expression levels of the genes in the different macrophage populations. Macro-1, 2, and 3, macrophages 1, 2 and 3 subpopulations.


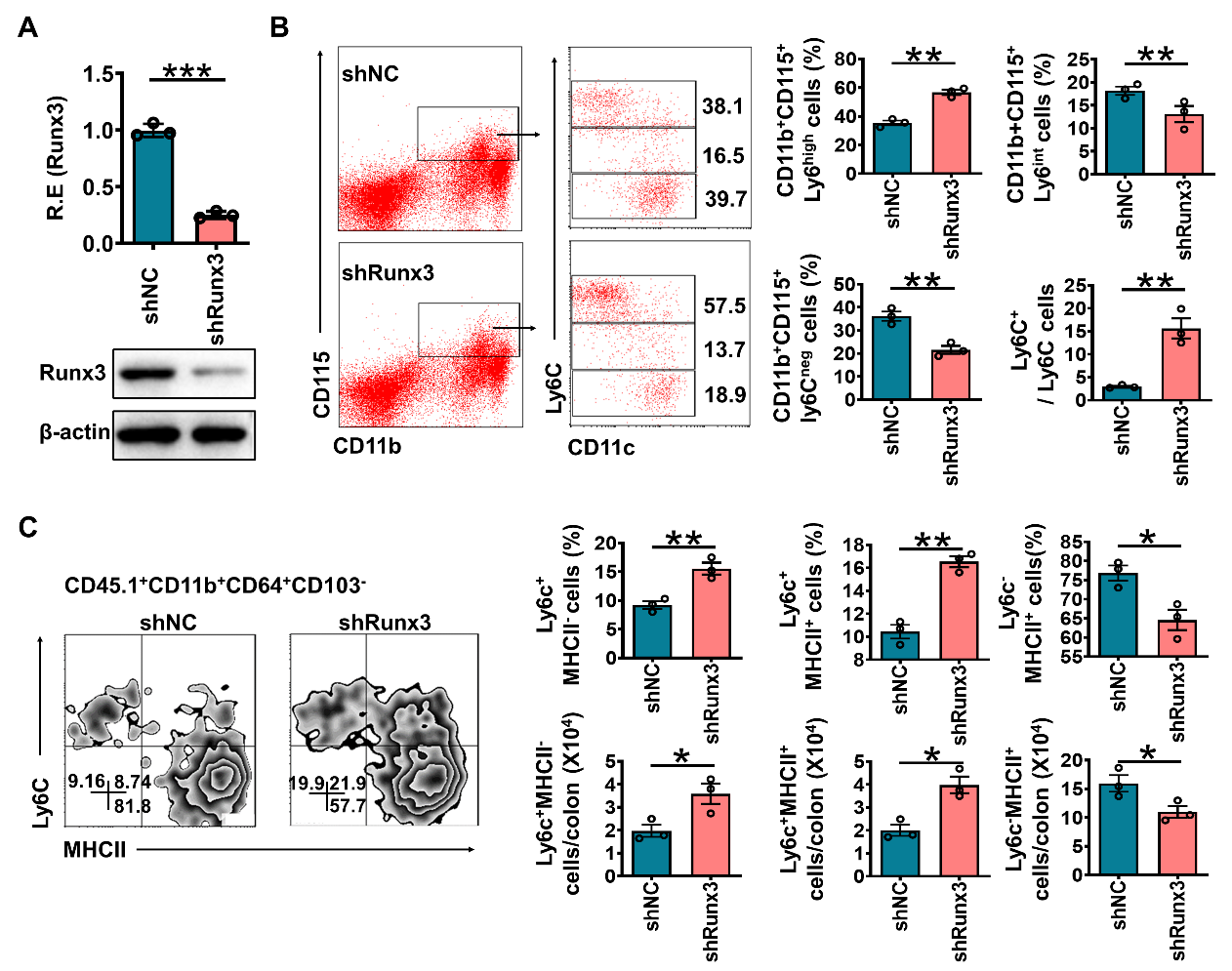


**Figure S2. Effects of silencing RUNX3 on the differentiation of Ly6C macrophages.** **A** qRT-PCR (upper) and immunoblot (lower) of *Runx3* in Runx3 shRNA lentiviruses (shRunx3) infected macrophages. R.E, relative expression. **B** Flow cytometry of CD11b^+^CD115^+^Ly6C^high^, CD11b^+^CD115^+^Ly6C^int^, CD11b^+^CD115^+^Ly6C^neg^ cells in peripheral blood of Runx3 shRNA lentiviruses (shRunx3) infected macrophages transplant model. shNC, control shRNA lentiviruses. % of Ly6C^high^, Ly6C^int^, and Ly6C^neg^ macrophages were compared (n=3). **B** Flow cytometry of CD45.1^+^CD11b^+^CD64^+^CD103^-^MHCǁ^+^Ly6C^+^ cell in colon tissues of Runx3 shRNA lentiviruses (shRunx3) infected macrophages transplant model. (n =3). shNC, control shRNA lentiviruses.

The analyses are based on a sample size of n=3 per group. Two side Student's t-test; **p<0.05,* ***p<0.01, ***p<0.001.* Data were a representative of at least three experiments.


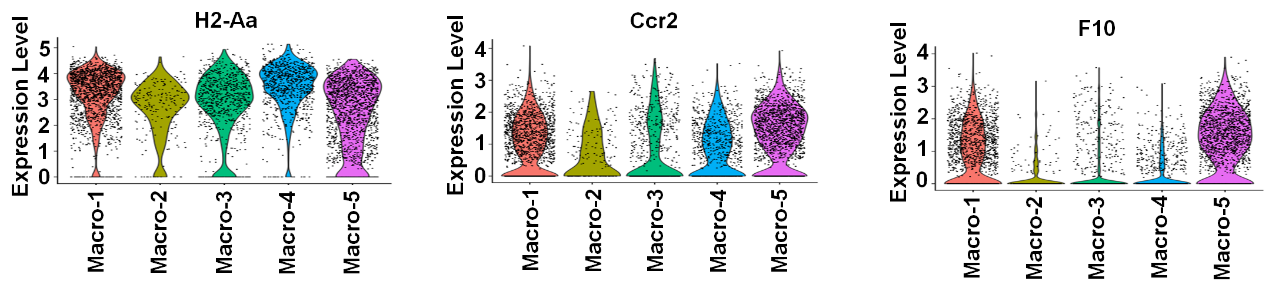


**Figure S3. Violin plots showing different expression levels of the genes in different macrophage subpopulations.** Macro-1- 5, 1-5 macrophage subpopulations.


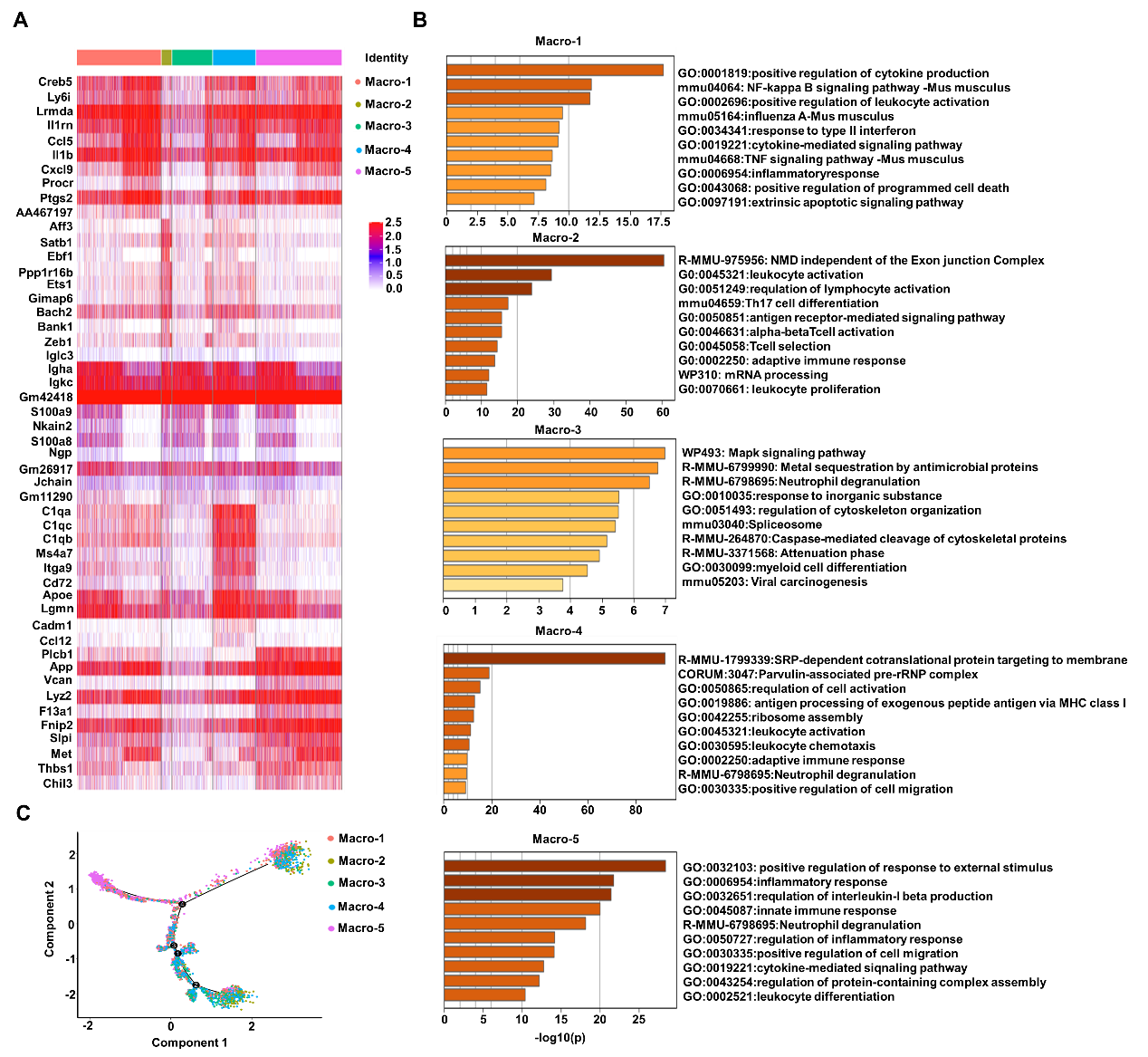


**Figure S4. Analyses of populations and subpopulations of macrophages from colon tissues. A** Heatmap of gene expression in different macrophage populations; **B** KEGG and GO analyses of different macrophage populations. **C** Monocle 2 trajectory analysis of different macrophage populations during their development.


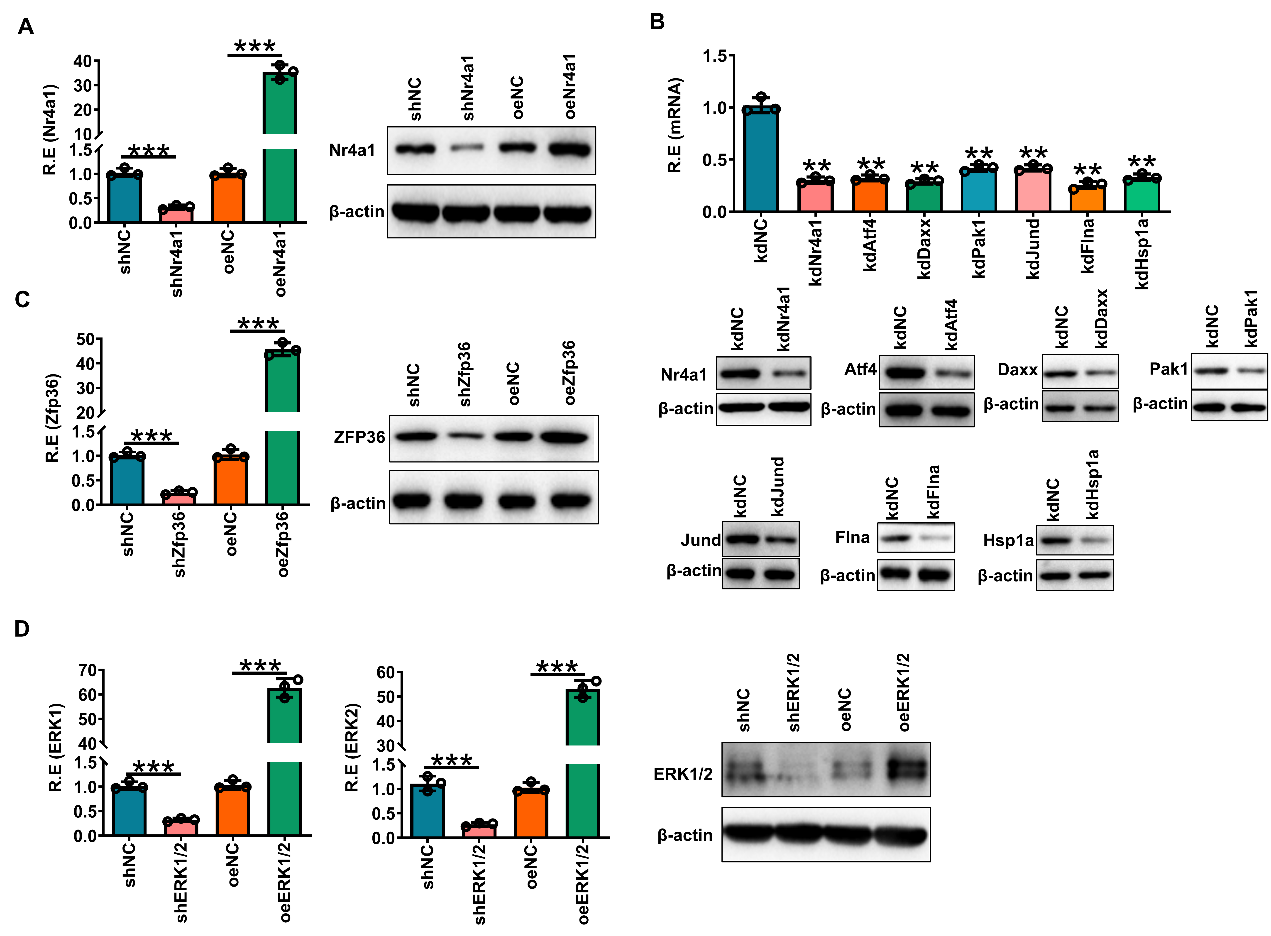


**Figure S5. The expression of genes in silencing and overexpressing lentivirus transfected macrophages.**

**A** qRT-PCR (right) and immunoblot (left) of Nr4a1 in Nr4a1 silencing (shNr4a1) or Nr4a1 overexpression (oeNr4a1) lentivirus transfected macrophages; **B** qRT-PCR (upper) and immunoblot (lower) of Nr4a1, Atf4, Daxx, Pak1, Jund, Flna and Hsp1a in different shRNA lentivirus transfected macrophages. KdNC, control shRNA lentiviruses; Kd, knockdown; **C** qRT-PCR (right) and immunoblot (left) of ZFP36 in ZFP36 silencing (shZFP36) or ZFP36 overexpression (oeZFP36) lentivirus transfected macrophages; **D** qRT-PCR (right) and immunoblot (left) of ERK1/2 in ERK1/2 silencing (shERK1/2) or ERK1/2 overexpression (oeERK1/2) lentivirus transfected macrophages. shNC, control shRNA lentiviruses; oeNC, control lentiviruses. The analyses are based on a sample size of n=3 per group. R.E, relative expression. Two side Student's t-test; *p<0.05, **p<0.01, ***p<0.001.


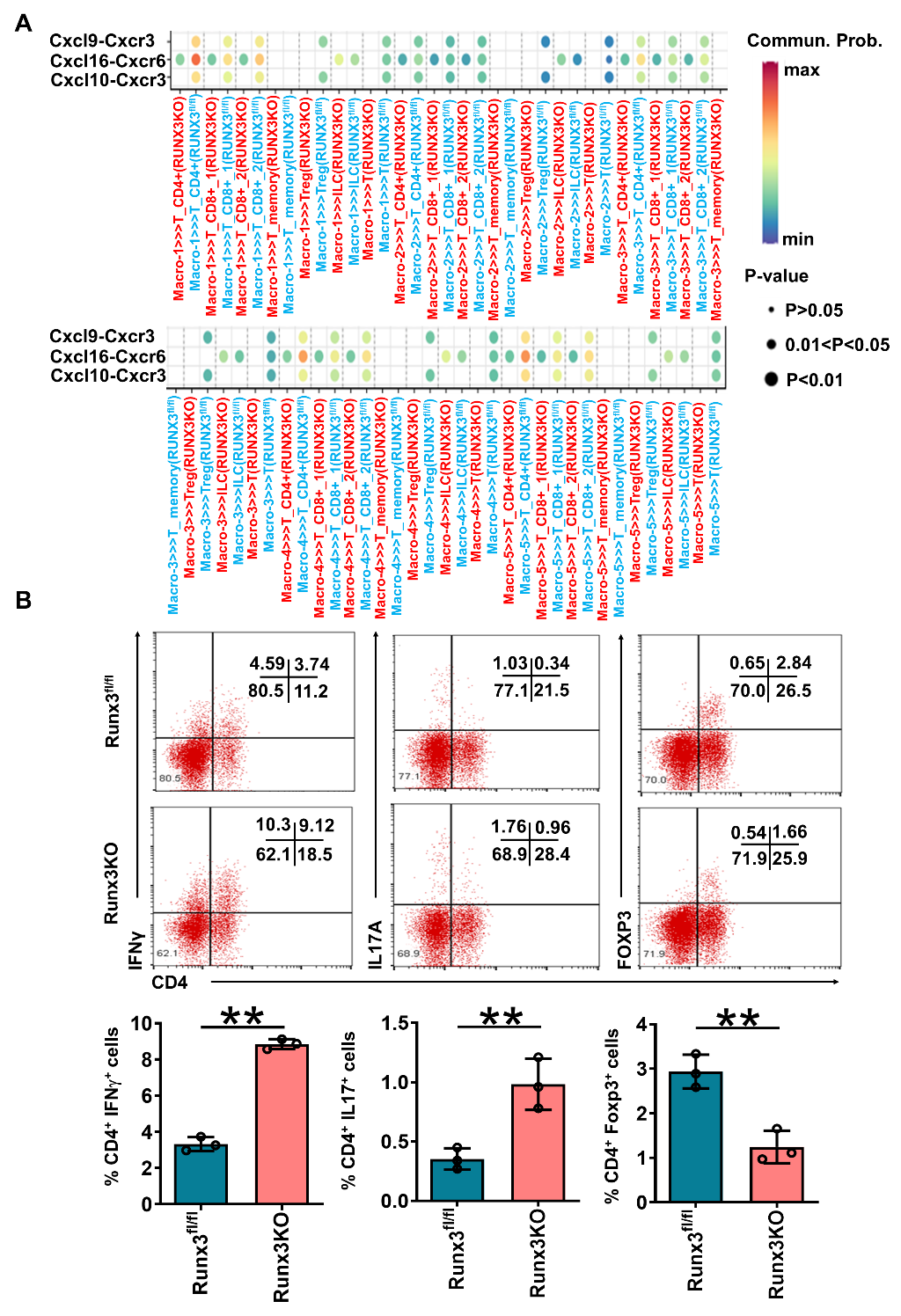


**Figure S6. Analyses between different macrophage populations and lymphocyte cells. A** Cellular communication analyses of different macrophage populations and lymphocyte cells. Macro-1- 5, macrophages 1-5 populations; **B** Flow cytometry analyses of Th1, Th17 and Treg cells in *RUNX3^fl/flpLuc-creT^* mice (RUNX3KO) and control *RUNX3*^fl/fl^ mice. The analyses in B are based on a sample size of n=3 per group. Two side Student's t-test; ***p<0.01.* Data were a representative of at least three experiments.

**
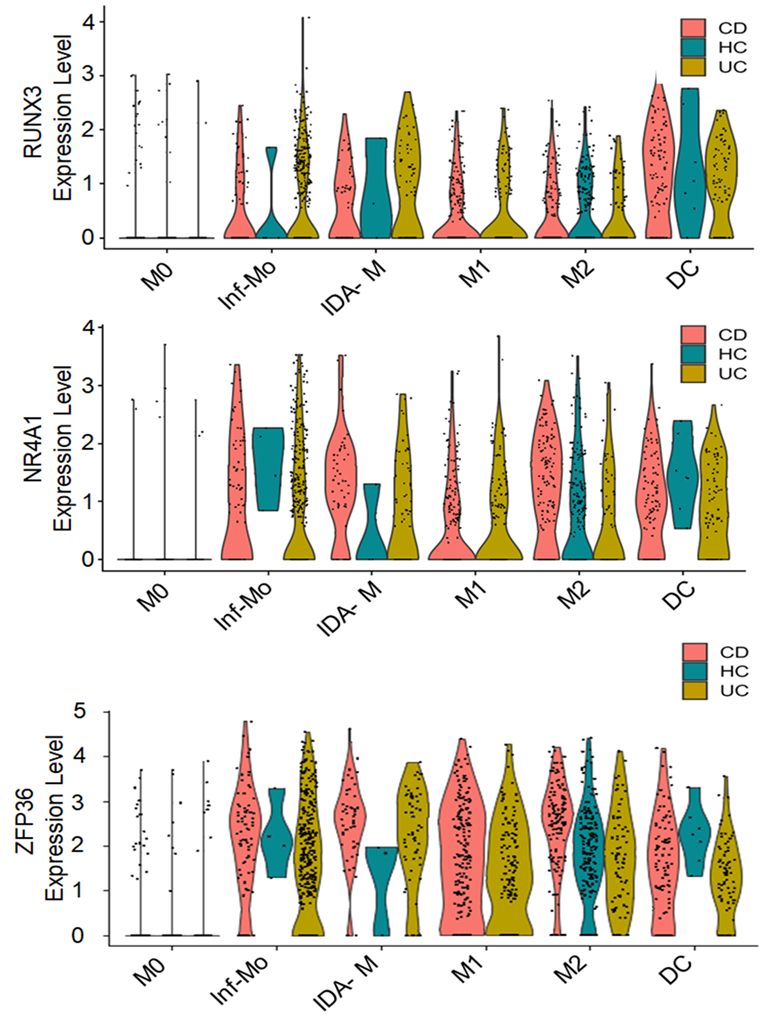
**

**Figure S7.** Violin plots of RUNX3, NR4A1 and ZFP36 in different macrophage populations of healthy individuals (HC), patients with CD (CD) or with UC (UC). M0, M0 macrophages; M2, M2 macrophages; DC, dendritic cells; M1, M1 macrophages; IDA-M, inflammation-dependent alternative macrophage and Inf-Mo, interferon monocytes.

**Table S1.** **| Reagents and oligoes used in this study.**

| REAGENT, RESOURCE OR OLIGOES | SOURCE | IDENTIFIER |
| --- | --- | --- |
| Antibodies | | |
| β-Actin Antibody | Santa Cruz | Cat:sc-47778 RRID: AB_626632 |
| Anti -IL1β antibody | Proteintech | Cat: 26048-1-AP RRID: AB_2880351 |
| Anti-Histone H3 (tri methyl K4) antibody | Abcam | Cat: ab8580 RRID: AB_306649 |
| C/EBPβ (LAP) Antibody | Cell Signaling Technology | Cat:3087S RRID: AB_2078052 |
| Anti-Nr4a1 Antibody | Proteintech | Cat: 12235-1-AP RRID: AB_10644125 |
| ATF4 Polyclonal antibody | Proteintech | Cat: 10835-1-AP RRID: AB_2058600 |
| DAXX Polyclonal antibody | Proteintech | Cat: 20489-1-AP RRID: AB_10693620 |
| PAK1 Polyclonal antibody | Proteintech | Cat: 21401-1-AP RRID: AB_11232232 |
| JunD Rabbit mAb | Cell Signaling Technology | Cat: 5000T RRID: AB_10949318 |
| Filamin A (FLNA) Rabbit mAb | Cell Signaling Technology | Cat: 4762S RRID: AB_2106408 |
| HSP70(HSPA1A) Polyclonal antibody | Proteintech | Cat: 10995-1-AP RRID: AB_2264230 |
| RUNX3/AML2 (D9K6L) antibody | Cell Signaling Technology | Cat: 13089 RRID: AB_2798118 |
| ZFP36 Monoclonal antibody | Proteintech | Cat: 66938-1-Ig RRID: AB_2882262 |
| F4/80 (6A545) antibody | Santa Cruz | Cat:sc-71085 RRID: AB_1122717 |
| p44/42 MAPK (Erk1/2) Antibody | Cell Signaling Technology | Cat: 9102 RRID: AB_330744 |
| Phospho-p44/42 MAPK (Erk1/2) (Thr202/Tyr204) Antibody | Cell Signaling Technology | Cat: 9101 RRID: AB_331646 |
| p38 MAPK Antibody | Cell Signaling Technology | Cat: 9212 RRID: AB_330713 |
| Phospho-p38 MAPK (Thr180/Tyr182) | Cell Signaling Technology | Cat: 4511 RRID:AB_2139682 |
| SAPK/JNK Antibody | Cell Signaling Technology | Cat: 9252 RRID:AB_2250373 |
| Phospho-SAPK/JNK (Thr183/Tyr185) Antibody | Cell Signaling Technology | Cat: 9251 RRID:AB_331659 |
| Erk5 Antibody | Cell Signaling Technology | Cat: 3372 RRID:AB_330491 |
| Phospho-Erk5 (Thr218/Tyr220) Antibody | Cell Signaling Technology | Cat: 3371 RRID: AB_2140424 |
| Anti-RbBP5 antibody | Abcam | Cat: ab52084 RRID: AB_882299 |
| WDR5 Rabbit mAb | Cell Signaling Technology | Cat:13105 RRID: AB_2620133 |
| Anti-ASH2L antibody | Abcam | Cat: ab50699 RRID: AB_867739 |
| Anti-DPY30 antibody | Abcam | Cat: ab126352 RRID: AB_11128034 |
| Anti-MLL1 (D2M7U) Rabbit antibody | Cell Signaling Technology | Cat:14689 RRID: AB_2688009 |
| Anti-CD11B (M1/70) antibody | Abcam | Cat: ab8878 RRID: AB_306831 |
| Recombinant Anti-IL-1 beta antibody | Abcam | Cat: ab 254360 RRID: AB_2936299 |
| Recombinant Anti-IL-1 alpha antibody | Abcam | Cat: ab300501 RRID: AB_2941899 |
| Anti-CCL4/MIP-1 beta antibody | Abcam | Cat: ab45690 RRID: AB_776129 |
| APC- anti-mouse CD45 30-F11 | Biolegend | Cat:103112 RRID: AB_312977 |
| FITC- anti-mouse CD11b M1/70 | eBioscience | Cat:11-0112-86 RRID: AB_464937 |
| APC anti-mouse CD11c Antibody | Biolegend | Cat: 117310 RRID: AB_ 313779 |
| PerCP anti-mouse/human CD11b Antibody | Biolegend | Cat: 135506 RRID: AB_1937253 |
| FITC anti-mouse Ly-6C Antibody | Biolegend | Cat: 128006 RRID: AB_ 1186135 |
| FITC-CD4 RM4-5 | BD Bioscience | Cat:561835 RRID: AB_10894386 |
| PE/Cyanine7 anti-mouse CD64 (FcγRI) Antibody | Biolegend | Cat: 164413 RRID: AB_ 3083142 |
| APC/Cyanine7 anti-mouse CD103 Antibody | Biolegend | Cat: 121431 RRID: AB_ 2566551 |
| PE anti-mouse CD115 (CSF-1R) Antibody | Biolegend | Cat: 165003 RRID: AB_ 2910350 |
| APC anti-mouse CD117 (c-Kit) Antibody | Biolegend | Cat: 105812 RRID:AB_ 313221 |
| PE anti-mouse I-A/I-E(MHC II) Antibody | Biolegend | Cat: 107607 RRID: AB_ 313322 |
| PE anti-mouse IFN-γ Antibody | Biolegend | Cat: 505807 RRID: AB_ 315401 |
| PE anti-mouse IL-17A Antibody | Biolegend | Cat: 506903 RRID: AB_ 315463 |
| PE anti-mouse FOXP3 Antibody | Biolegend | Cat: 126403 RRID: AB_ 1089117 |
| Chemicals, Peptides, and Recombinant Proteins | | |
| Recombinant Murine GM-CSF | PeproTech | Cat:315-03 |
| Recombinant Murine M-CSF | PeproTech | Cat:315-02 |
| Recombinant Human M-CSF | PeproTech | Cat: 300-25 |
| Trizol | Life technologies | Cat:15596018 |
| ERK1/2 inhibitor | MCE | Cat: HY-112287 |
| Oligonucleotides for qRT-PCR | | |
| Murine GAPDH FW | BGI | 5’-TCAACGGCACAGTCAAGG-3’ |
| Murine GAPDH REV | BGI | 5’-TACTCAGCACCGGCCTCA-3’ |
| Murine LncLy6C FW | BGI | 5’-CTTAATGTTTCTCTTTCTGGG-3’ |
| Murine LncLy6C REV | BGI | 5’-TTTCTTTAGGTCTGTTGCTGA-3’ |
| Murine IL1β FW | BGI | 5’-TCGCAGCAGCACATCAACAAG-3’ |
| Murine IL1β REV | BGI | 5’-GAAGGTCCACGGGAAAGACAC-3’ |
| Murine RUNX3 FW | BGI | 5’-CGGCAAGATGGGCGAGAACA-3’ |
| Murine RUNX3 REV | BGI | 5’-GGCAGCACGGAGCAGAGGAA-3’ |
| Murine ZFP36 FW | BGI | 5’-TGTCCTCTTGTTCCTTTTCG-3’ |
| Murine ZFP36 REV | BGI | 5’-GGCCTGGTTAGGGTCTCTTC-3’ |
| Murine IL-6 FW | BGI | 5’-ACAACCACGGCCTTCCCTACTT-3’ |
| Murine IL-6 Rev | BGI | 5’-TTTCTCATTTCCACGATTTCCC-3’ |
| Murine IL-10 FW | BGI | 5’-ACAACATACTGCTAACCGAC-3’ |
| Murine IL-10 Rev | BGI | 5’-TTCTCACCCAGGGAATTCAA-3’ |
| Murine TGFβ FW | BGI | 5’-GAAGGACCTGGGTTGGAAGT-3’ |
| Murine TGFβ Rev | BGI | 5’-CGGGTTGTGTTGGTTGTAGA-3’ |
| Murine TNFa FW | BGI | 5’-CCAGACCCTCACACTCAGATCA-3’ |
| Murine TNFa Rev | BGI | 5’-GTAGACAAGGTACAACCCATCG-3’ |
| Murine CCL5 FW | BGI | 5’-GCTGCTTTGCCTACCTCTCC -3’ |
| Murine CCL5 Rev | BGI | 5’-TCGAGTGACAAACACGACTGC-3’ |
| Murine Nr4A1 Fw | BGI | 5’-TGAGTTCGGCAAGCCTACCAT-3’ |
| Murine Nr4A1 Rev | BGI | 5’-GGAGGAGGCAGAGGAACAAGC-3’ |
| Murine Hspa1a FW | BGI | 5’- CCACCATCCCCACCAAGCAG -3’ |
| Murine Hspa1a REV | BGI | 5’- AGTTCGAAGCGCCCCAGCAG -3’ |
| Murine Flna FW | BGI | 5’- ACCTACCTGTCTCAGTTTCC -3’ |
| Murine Flna REV | BGI | 5’- ATTCTGCTCTCTTCTTCACC -3’ |
| Murine Jund FW | BGI | 5’- GCGCAAGCTGGAGCGTATCT -3’ |
| Murine Jund REV | BGI | 5’- TGTTGACGTGGCTGAGGACT -3’ |
| Murine Pak1 FW | BGI | 5’- GAGAATAACACCACTCCGCC -3’ |
| Murine Pak1 REV | BGI | 5’- CACACTCACTATGCTCCGTA -3’ |
| Murine Daxx FW | BGI | 5’- ATTCCCTCCCCACCATCTTG -3’ |
| Murine Daxx REV | BGI | 5’- CTGTGCCATCGGTTCTTTTA -3’ |
| Murine Atf4 FW | BGI | 5’- GAGTCCTACCTGGGCTCTCC -3’ |
| Murine Atf4 REV | BGI | 5’- GGCTGCTGTCTTGTTTTGCT -3’ |
| Murine WDR5 FW | BGI | 5’- AAAACTTTGAAGATTTGGGA -3’ |
| Murine WDR5 REV | BGI | 5’- TGAGACGATGAGGTTGGACT -3’ |
| Murine RBBP5 FW | BGI | 5’- GGACAAGCAATACTACCGCC -3’ |
| Murine RBBP5 REV | BGI | 5’- CTCTCCATCCCTTCCACACG -3’ |
| Murine ASH2L FW | BGI | 5’- GCACTATTCGTCTGGCTACG -3’ |
| Murine ASH2L REV | BGI | 5’- TCACTATGGGGGGTCTGTTT -3’ |
| Murine MLL FW | BGI | 5’- GAAGAAGTCAGAGTGCGAAG -3’ |
| Murine MLL REV | BGI | 5’- AGAGGGGAAAACACAGATGG -3’ |
| Murine DPY30 FW | BGI | 5’-CAGGGACTTGCTGTGCTTGC -3’ |
| Murine DPY30 REV | BGI | 5’- AACTCGATGGGATTTGGTGG -3’ |
| Murine Erk1 FW | BGI | 5’-AGATCTGTGATTTTGGCCTG -3’ |
| Murine Erk1 REV | BGI | 5’- GTCGATGGATTTGGTGTAGC -3’ |
| Murine Erk2 FW | BGI | 5’-ACTATTTGCTTTCTCTCCCG -3’ |
| Murine Erk2 REV | BGI | 5’- CTGTTCAACTTCAATCCTCT -3’ |
| Human GAPDH FW | BGI | 5’-TCAAGAAGGTGGTGAAGCAGG-3’ |
| Human GAPDH REV | BGI | 5’- AGCGTCAAAGGTGGAGGAGTG-3’ |
| Human LncLy6C FW | BGI | 5’-ATTCATTACCTTTTCTTTGCTC-3’ |
| Human LncLy6C REV | BGI | 5’-CAGTGTTTGATTTTTACAGTCC-3’ |
| shRNAs/siRNAs used in this study | | |
| Murine RUNX3 | Ribobio | 5’-AGACUCAGCAGCCUGGAUGAA-3’ |
| Murine Hspa1a | Ribobio | 5’-AGAAGAAGGUGCUGGACAAGU-3’ |
| Murine Flna | Ribobio | 5’-CAGUCAGUGUCAAGUACAAGG-3’ |
| Murine Jund | Ribobio | 5’-CGACGAGCAGCAUGCUGAAGA-3’ |
| Murine Pak1 | Ribobio | 5’-GGCACAGUGUAUACUGCAAUG-3’ |
| Murine Daxx | Ribobio | 5’-AGAUCUAUGUGUACAUUAACG-3’ |
| Murine Atf4 | Ribobio | 5’-GGAGUUAGUUUGACAGCUAAA-3’ |
| Murine Nr4a1 | Ribobio | 5’-GGACAGAGCAGUUGCCUAAGG-3’ |
| Murine ZFP36 | Ribobio | 5’-GGAGGACUUUGGAACAUAAAC-3’ |
| Murine WDR5-1 | Ribobio | 5’- GCGUGGUCAUCAGAUUCUAAC -3’ |
| Murine WDR5-2 | Ribobio | 5’- GAUUGUGUCUGGUUCUGAAGA -3’ |
| Murine RBBP5-1 | Ribobio | 5’-GGGCUAGUUCAGAGAAGAAGA -3’ |
| Murine RBBP5-2 | Ribobio | 5’- GGAAGAUUCAAAGGCUCUAUU -3’ |
| Murine ASH2L-1 | Ribobio | 5’- GAGAUGUGUUCUUGGUAAAGG -3’ |
| Murine ASH2L-2 | Ribobio | 5’- CCAUGUUCUCCAAAGAUAAGG -3’ |
| Murine MLL-1 | Ribobio | 5’-GGAUCAGAGUGGACUUUAAGG -3’ |
| Murine MLL-2 | Ribobio | 5’- GGAAGUCACUGACAGAUAAAG -3’ |
| Murine DPY30-1 | Ribobio | 5’- GACAGUUGUGCCUAUCUUAUU -3’ |
| Murine DPY30-2 | Ribobio | 5’- GCGUUGAGAGAAUAGUCGAAA -3’ |
| Murine Erk1 | Ribobio | 5’- GAGAGAUGUUUACAUUGUUCA-3’ |
| Murine Erk2 | Ribobio | 5’- GGAAGAUCUGAAUUGUAUAAU-3’ |
| P1 FW | BGI | 5’-GTGGCTTTTGTCTCTATTCGTG-3’ |
| P1 REV | BGI | 5’-GCCTATATCGGGTCATTTGTTG-3’ |
| Other |  |  |
| DSS | MP Biomedicals | Cat: 0216011080 |
| DMEM | Gibco | Cat: 11965118 |
| FBS | Gibco | Cat:10099141 |
| HBSS | Gibco | Cat:14170161 |
| Percoll | Solarbio | Cat: P8370 |
| Mouse IL1β ELISA Kit | abclone | Cat: RK04878 |
| Mouse CCL5 ELISA Kit | ThermoFisher | Cat: BMS6009INST |
| Human Butyric acid (N-BA) ELISA Kit | COIBO BIO | Cat: CB13910-Hu |
| EZ-ChIP™Chromatin Immunoprecipitation Kit | Millipore | Cat:17-371 |
